# Biomaterials Facilitating Dendritic Cell‐Mediated Cancer Immunotherapy

**DOI:** 10.1002/advs.202301339

**Published:** 2023-04-23

**Authors:** Heng Dong, Qiang Li, Yu Zhang, Meng Ding, Zhaogang Teng, Yongbin Mou

**Affiliations:** ^1^ Nanjing Stomatological Hospital Affiliated Hospital of Medical School, Nanjing University 30 Zhongyang Road Nanjing Jiangsu 210008 P. R. China; ^2^ Key Laboratory for Organic Electronics and Information Displays Jiangsu Key Laboratory for Biosensors Institute of Advanced Materials Jiangsu National Synergetic Innovation Centre for Advanced Materials Nanjing University of Posts and Telecommunications 9 Wenyuan Road Nanjing Jiangsu 210023 P. R. China

**Keywords:** biomaterials, cancer immunotherapy, dendritic cells, nanovaccine, tumor microenvironment

## Abstract

Dendritic cell (DC)‐based cancer immunotherapy has exhibited remarkable clinical prospects because DCs play a central role in initiating and regulating adaptive immune responses. However, the application of traditional DC‐mediated immunotherapy is limited due to insufficient antigen delivery, inadequate antigen presentation, and high levels of immunosuppression. To address these challenges, engineered biomaterials have been exploited to enhance DC‐mediated immunotherapeutic effects. In this review, vital principal components that can enhance DC‐mediated immunotherapeutic effects are first introduced. The parameters considered in the rational design of biomaterials, including targeting modifications, size, shape, surface, and mechanical properties, which can affect biomaterial optimization of DC functions, are further summarized. Moreover, recent applications of various engineered biomaterials in the field of DC‐mediated immunotherapy are reviewed, including those serve as immune component delivery platforms, remodel the tumor microenvironment, and synergistically enhance the effects of other antitumor therapies. Overall, the present review comprehensively and systematically summarizes biomaterials related to the promotion of DC functions; and specifically focuses on the recent advances in biomaterial designs for DC activation to eradicate tumors. The challenges and opportunities of treatment strategies designed to amplify DCs via the application of biomaterials are discussed with the aim of inspiring the clinical translation of future DC‐mediated cancer immunotherapies.

## Graphical abstract

Biomaterial‐mediated dendritic cell (DC) immunotherapy aims to boost the patient's own immune response against tumors. Recent advances in the applications of various engineered biomaterials in the field of DC immunotherapy are comprehensively summarized here. Functional biomaterials can serve as immune component delivery platforms, remodel the tumor microenvironment, and synergistically enhance the effects of other antitumor therapies.

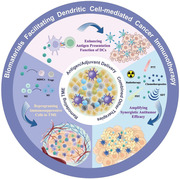

## 1 Introduction

Dendritic cells (DCs) are important in innate and adaptive immune responses. Specifically, DCs can present tumor antigens and express highly costimulatory molecules to effectively activate cytotoxic T lymphocytes (CTLs) for cancer immunotherapy.^[^ ^1^ ^]^ To elicit effective CTL responses, immature DCs (iDCs) uptake, process, and present antigens to major histocompatibility complex (MHC) molecules on their surface. The antigens bound to MHC molecules activate naïve T (Tn) cells into the CTLs.^[^ ^2^ ^]^ For antigen binding, iDCs are primed to differentiate into mature DCs (mDCs) via phenotypic and functional transformations. The transformations are enhanced and adjusted by a variety of receptors,^[^ ^3^ ^]^ including chemokine receptors, adhesion receptors, and costimulatory molecules. Moreover, MHC molecules on mDCs are also highly expressed to optimally activate antitumor immune responses.^[^ ^4^ ^]^

Although DC‐mediated immunotherapies are considered to be effective options in immunotherapy, traditional DC vaccines are hampered by a variety of limitations. For instance, Sipuleucel‐T was the first DC‐mediated vaccine approved by the US Food and Drug Administration (FDA) in 2010. For the vaccine, mDCs are isolated from patients' peripheral blood, cultured to maturity, loaded with antigen, and infused back into the patient to induce tumor‐specific immune responses.^[^ ^5^ ^]^ However, the preparation process of Sipuleucel‐T is time‐consuming and laborious. In addition, simple DC‐mediated vaccines generally cannot effectively activate the antitumor response in vivo due to insufficient antigen delivery, inadequate antigen presentation, and the immunosuppressive tumor microenvironment (TME).^[^ ^6^ ^]^ The immunosuppressive TME inhibits the initiation of tumor‐infiltrated DCs to impair the antitumor T‐cell response, resulting in tumor progression, metastasis, and poor prognosis.^[^ ^7^ ^]^

Engineered biomaterials have been designed and explored for promoting DC‐mediated immunotherapy.^[^ ^8^ ^]^ Engineered biomaterials, including nanocarriers, microcarriers, microneedles, and 3D scaffolds, etc., have exhibited several advantages in optimizing DC‐mediated immunotherapy.^[^ ^9^ ^]^ To achieve better DC‐mediated antitumor efficacy, the parameters of biomaterials, such as size, shape, specific surface area, mechanical properties, surface modifications, and carrying capacity, can be designed to promote antigen presentation and enhance the secretion of appropriate immune‐stimulatory molecules from DCs.^[^ ^10^ ^]^ In addition, when their physicochemical properties are optimized, the functionally engineered biomaterials themselves can be used as adjuvants for priming the maturation and migration of DCs or as delivery platforms for various kinds of molecules. Biomaterials can also protect against the degradation of antigenic peptides or agonists in blood or intracellular spaces. Within DCs, loaded cargo can be controlled released through the modification of chemically reactive components on the biomaterial surface, which enhances antigenic presentation. Rational design and modification of biomaterials to activate robust DC‐mediated immune responses are, therefore, of vital importance.

**Figure 1:**
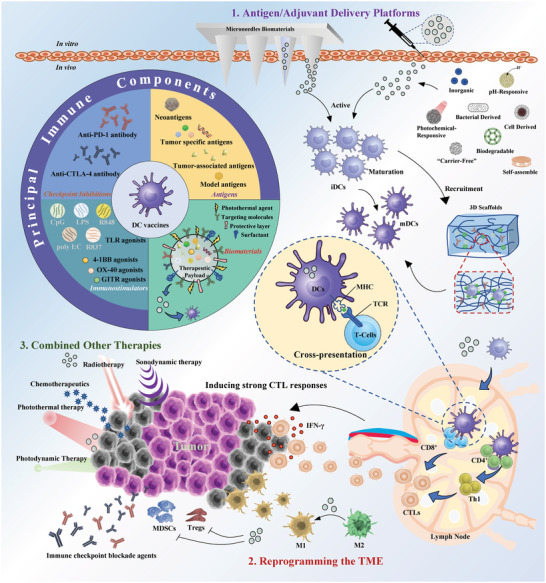
Schematic illustration of engineered biomaterials amplifying DCs to elicit antitumor immunity. (1) Biomaterials as delivery platforms, can carry a variety of immune components, such as tumor antigens and immunostimulators. Different types of biomaterials can mature iDCs into mDCs and activate the antigen‐presentation pathway of DCs to induce the CTL responses. Biomaterial‐mediated DC activation can combine immune checkpoint blockade agents (ICBs) to overcome immunosuppression in the TME. (2) In the TME, some biomaterials alter the functions of immunosuppressive cells, such as myeloid‐derived suppressor cells (MDSCs), regulatory T cells (Tregs), and tumor‐associated macrophages (TAMs). (3) Biomaterials combined with other therapies (e.g., radiotherapy, chemotherapy, photothermal therapy, photodynamic therapy, or sonodynamic therapy) can synergistically promote DC‐related antitumor immunotherapies and empower tumor eradication.

The interactions between biomaterials and DCs in immunotherapy have attracted increasing interest from many researchers.^[^ ^11^ ^]^ With advances and continuous evolution of biomaterials science, many biomaterials have been explored and applied in DC‐mediated tumor immunotherapy (**Figure** **1**). In this review, we first summarize current barriers of traditional DC‐based vaccines and summarize the principal components for promoting DC antitumor functions, such as antigens, immunostimulators, and immune checkpoint blockade agents. We further cover the general effects of ligand, size, shape, surface, and mechanical properties of engineered biomaterials in the stimulation of DCs. Moreover, we summarize diverse biomaterials for antigen/adjuvant delivery, including non‐degradable biomaterials, biodegradable biomaterials, self‐assembly biomaterials, naturally‐derived biomaterials, stimulated responsive biomaterials, “carrier‐free” biomaterials, microneedles, and 3D scaffolds. In addition, biomaterials facilitating DC effects based on overcoming obstacles of the TME are described, e.g., biomaterials modulating myeloid‐derived suppressor cells (MDSCs), regulatory T cells (Tregs) and promoting M1 polarization of tumor‐associated macrophages (TAMs) in the TME. Recent advances concerning combinate biomaterial‐mediated DC immunotherapy with other therapies, including radiotherapy, chemotherapy, photodynamic therapy (PTT), photodynamic therapy (PDT), and sonodynamic therapy (SDT), are also reviewed as potential therapeutic strategies. Finally, challenges and future opportunities for the biomedical applications of DC immunotherapy are also discussed. The present review provides necessary foundations and considerations for the exploitation of biomaterials for more effective and large‐scale DC‐mediated cancer immunotherapy. Meanwhile, we highlight the unique properties and roles of different biomaterials in DC‐mediated immunotherapy as well as potential future applications.

## 2 Current Barriers of Traditional DC‐Based Vaccines

Antigen presentation is a crucial process for immunotherapy that includes antigen internalization, protein degradation, and loading of antigenic peptides onto MHC molecules of antigen‐presenting cells (APCs).^[^ ^12^ ^]^ DCs are the most important APCs, with the ability to present exogenous antigens.^[^ ^13^ ^]^ Specifically, DCs sample and load antigenic peptides onto both MHC‐I molecules via a cross‐presenting pathway, which is critical in the induction of adaptive immune responses to suppress tumors.^[^ ^14^ ^]^ Antigenic peptides bound to MHC‐I on DC surfaces are then recognized by T‐cell receptors (TCRs), which prime and activate CD8^+^ T cells. CD8^+^ cytotoxic T lymphocytes (CTLs) are well‐known key producers of interferon‐*γ* (IFN‐*γ*) for antitumor immunity.^[^ ^15^ ^]^ As the ultimate effector T cells, CTLs are activated by DCs through the cross‐presentation pathway.^[^ ^16^ ^]^ Therefore, the intensity and duration of interactions between DCs and T cells affect the T‐cell activation process. Additionally, the upregulation of costimulatory molecules on DC surfaces is pivotal in promoting and maintaining long‐term and stable contact with T cells.^[^ ^17^ ^]^ Thus, it is important to build effective intercellular communication between DCs and T cells to effectively elicit antitumor immune responses.

**Figure 2:**
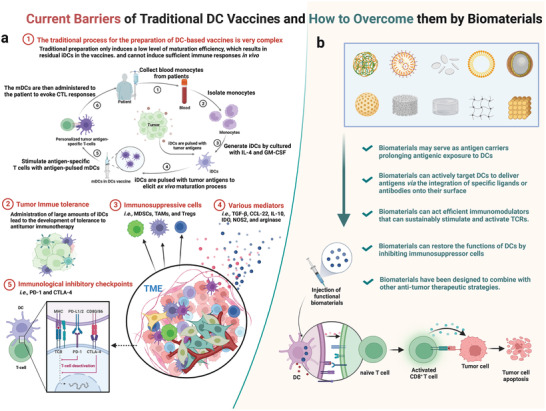
Current barriers of traditional DC vaccines and how to overcome them by biomaterials. a) Schematic illustration of current limitations of traditional DC vaccines. b) Some biomaterials have been utilized to promote DC‐mediated antitumor immune response.

DCs are a central platform for activating T cells in immunotherapy because DCs have favorable biosecurity and intrinsic capacities for enhancing immune responses against tumor cells.^[^ ^17^, ^18^ ^]^ However, there are some limitations in traditional DC vaccine treatment (**Figure** **2**a). For example, to obtain a large number of DCs as vaccines for the production of CTLs, *ex vivo* culture systems were invented at the end of the 20th century.^[^ ^19^ ^]^ Traditionally, the preparation of DC‐based vaccines includes three main steps: 1) blood monocytes are cultured with interleukin‐4 (IL‐4) and granulocyte‐macrophage colony‐stimulating factor (GM‐CSF) to generate a uniform population of iDCs; 2) iDCs are pulsed with tumor antigens to elicit ex vivo maturation process; and 3) mDCs are then administered to the patient to evoke CTL responses.^[^ ^20^ ^]^ However, the traditional process for the preparation of DC‐based vaccines is very complex and only induces a low level of maturation efficiency, which results in residual iDCs in the vaccines. The administration of large amounts of iDCs still cannot induce sufficient immune responses in vivo.^[^ ^21^ ^]^ This leads naïve T cells differentiate into Tregs or other suppressive effector T cells. These adverse effects lead to the development of tolerance to antitumor immunotherapy.^[^ ^22^ ^]^ In contrast, mDCs can induce IFN‐*γ* secretion by functionally superior CD8^+^ T cells.^[^ ^23^ ^]^ Moreover, functional maturation of DCs enhances their migration from the site of administration to T‐cell enriched draining lymph nodes (dLNs) and then promotes effective intercellular communication of DCs and T cells.^[^ ^24^ ^]^ Therefore, efficacious strategies are urgently needed to promote DC maturation in DC vaccines.

In addition, an important reason for the inability to obtain adequate mDCs from cancer patients is the multiplex immunosuppressive mechanisms in the TME.^[^ ^25^ ^]^ The activation, maturation, and differentiation of iDCs can be suppressed or altered by immunosuppressive cells, including MDSCs, tumor‐associated macrophages (TAMs), and Tregs, which secrete various mediators, including transforming growth factor‐*β* (TGF‐*β*), C‐C motif chemokine 22 ligands (CCL‐22), interleukin‐10 (IL‐10), indoleamine 2,3‐dioxy‐genase (IDO), nitric oxide synthase 2 (NOS2), and arginase, that create an immunosuppressive TME for tumorigenesis.^[^ ^26^ ^]^ Some immunological inhibitory checkpoints, such as cytotoxic T lymphocyte antigen‐4 (CTLA‐4) and programmed cell death protein‐1 (PD‐1), which are highly expressed on T cells, also hinder the effective interactions between T cells and DCs. For example, CTLA‐4 competitively binds to costimulatory molecules on mDCs and thereby prevents antigenic presentation to T cells. This effect inhibits the priming of naïve T cells in lymphoid organs.^[^ ^27^ ^]^ When mDCs initiate T‐cell activation, the surface molecule programmed death‐ligand 1 (PD‐L1)/PD‐L2 on tumor cells engages the PD‐1 receptor on T cells, which suppresses the cytokine production, expansion, and cytolytic function of T cells. Moreover, PD‐L1 on tumor cells, DCs, or other cells in the TME inhibits proliferation and cytokine production by PD‐1‐positive T cells, leading to anergy and exhaustion of effector T cells.^[^ ^28^ ^]^

Although many clinical trials that have utilized DC‐based vaccines in humans have exhibited an absence of significant toxicity, the clinical outcomes and antitumor immune responses have been limited due to the above challenges.^[^ ^29^ ^]^ It is important to devise ways to overcome the above obstacles to improve the clinical efficacies of DC‐based vaccines. In this regard, some biomaterials have been utilized due to their unique properties that are essential in DC‐mediated immunity (Figure 2b). These biomaterials have superior active functions, such as serving as antigen carriers that prolong antigenic exposure to DCs. In addition, they can actively target DCs to deliver antigens via the integration of specific ligands or antibodies onto their surface. In particular, biomaterials are also efficient immunomodulators that can sustainably stimulate and activate TCRs. Biomaterials have also been designed to restore the functions of DCs by inhibiting immunosuppressor cells or coordinating multiple antitumor therapeutic strategies.

## 3 Potential Components for Promoting Biomaterial‐Mediated DC Activation

Antigen presentation is a crucial process for immunotherapy. DCs are highly specialized to take up and process antigens to induce CTL responses. Activating effective antitumor immunity by DCs requires appropriate antigen components. In addition, mDCs loaded with antigens are a prerequisite to induce therapeutic and protective antitumor immunity.^[^ ^30^ ^]^ To gain enough mDCs, immunostimulators (e.g., Toll‐like receptor agonists) are also important for activating the maturation of iDCs.^[^ ^31^ ^]^ Furthermore, DC‐mediated immunotherapy needs to either enhance DC functions or relieve the impact of the TME. The application of immune checkpoint blockade agents (ICBs) can reduce tumor‐elicited immune suppression by modulating the TME.^[^ ^32^ ^]^

**Figure 3:**
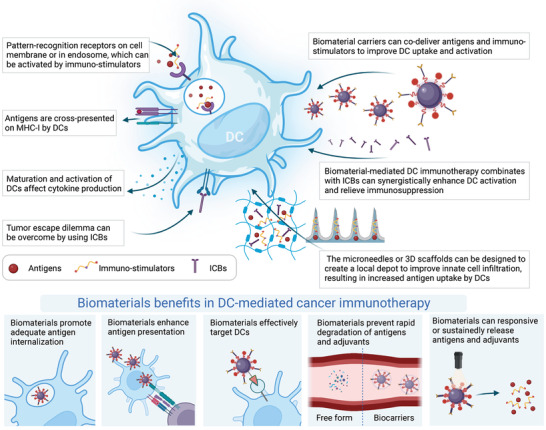
Biomaterials enhance DC activation in cancer immunotherapy. Biomaterials, such as nano/microparticles, microneedles and scaffolds, can be used as vehicles for the controlled delivery of antigens and adjuvants and interact with the immune system in a spatiotemporally controlled manner. These biomaterials can be designed to enhance DC activation. Antigens and adjuvant molecules can be codelivered to improve DC recognition and uptake of immune components. Particle‐form biomaterials can further improve endocytosis by DCs. 3D scaffolds can create a local depot to improve immune cell infiltration, resulting in increased antigen uptake and presentation. Biomaterial‐mediated DC activation can increase cytokine and chemokine production to improve antitumor immune responses. In addition, biomaterials can effectively target DCs by modifying targeting ligands. Biomaterials prevent rapid degradation of antigens and adjuvants in blood and lymphatic vasculature. Biomaterials can also responsive or sustainedly release antigens and adjuvants.

The commencement of the biomedical engineering era has brought exhilarating opportunities in the fields of biomaterials delivering immune components (**Figure** **3**). Protein‐, peptide‐, DNA‐, and mRNA‐based free antigens result in poor immunological responses due to inadequate internalization and rapid degradation. Advantages of biomaterials lie in their ease of internalization, enhanced antigen presentation, effective targeting property, and renal filtration escape.^[^ ^33^ ^]^ Therefore, antigens can be incorporated into biomaterials to overcome the above drawbacks. Biomaterials have been designed to protect antigens from degradation and increase the duration of immune responses. Moreover, decoration of immune adjuvants and tumor antigens on the nanocarrier surface could help boost immunity during circulation. TLR ligands are well known for their ability to induce DC maturation and have direct effects on promoting cross‐presentation by DCs and potent cellular immunity.^[^ ^34^ ^]^ However, rapid degradation and ineffective delivery into intracellular compartments are major obstacles. For example, the TLR9 ligand CpG has potent immunostimulatory adjuvant activity, and CpG needs to be internalized due to intracellular localization of TLR9.^[^ ^35^ ^]^ Antigens and CpG must be colocalized in the same DC to generate the most potent therapeutic antigen‐specific immune responses.^[^ ^36^ ^]^ Combining TLR ligands and antigens in the same biomaterial carrier is more potent than separate administration, which protects CpG from enzymatic degradation, improves uptake by DCs and targeting of CpG to the endolysosomes, and ensures codelivery of antigens and CpG to the same DCs.^[^ ^37^ ^]^ In addition, incorporation of antigens and adjuvants within engineered biomaterials allows for site‐specific immunization, responsive or sustained release, and protection from degradation during circulation. Thus, the addition of a TLR ligand as an adjuvant to biomaterials is a promising treatment strategy to maximally induce enhanced cross‐presentation by DCs. ICB therapies are effective in various types of tumors, but the response rate depends on pre‐existing immunity.^[^ ^38^ ^]^ ICB treatment by triggering nonspecific immune responses still possesses side effects and appears to be effective for fewer than 20% of tumor patients in clinical studies.^[^ ^39^ ^]^ Moreover, ICB therapy alone did not confer significant tumor growth control for aggressive tumors due to limited immune responses.^[^ ^40^ ^]^ It has been reported that biomaterial‐mediated DC immunotherapy in further combination with ICBs can inhibit tumor metastases and prevent tumor relapse by synergistically enhancing immune activation and relieving immunosuppression.^[^ ^41^ ^]^ Therefore, it is hoped that the combination of ICBs with a biomaterial‐enhanced DC‐activated strategy may bring new fortune for tumor therapy. Representative combinations of different components with biomaterials relevant to DCs are described as follows in **Table** **1**.

**Table 1:** Representative biomaterial‐based strategies for promoting DC‐based immunotherapy (Abbreviations: OVA, ovalbumin; CpG, CpG oligonucleotide ligands; PEI, polyethyleneimine; GM‐CSF, granulocyte‐macrophage colony‐stimulating factor; PLGA, poly(d,l‐lactide‐co‐glycolide); R837, imiquimod; *α*PD‐1, antibody of programmed cell death protein 1; *α*CTLA‐4, antibody of cytotoxic T lymphocyte antigen‐4; ICBs, immune checkpoint blockade agents; N/A, not applicable)

No. ^[Ref.]^	Biomaterials	Antigens	Immuno‐stimulators	ICBs	Tumor model
1[42]	AlO(OH)‐polymer nanoparticles	OVA antigen, B16F10 lysates	CpG	N/A	B16‐OVA, B16F10
2[40]	PEI‐absorbed mesoporous silica microrods	Neoantigen peptide	CpG, GM‐CSF	*α*CTLA‐4	B16F10, TC‐1
3[41a]	Synthetic high‐density lipoprotein nanodiscs	OVA_257‐264_ peptide, Neoantigen Adgpk	CpG	*α*PD‐1 and *α*CTLA‐4	MC‐38, B16F10
4[43]	Adjuvant‐loaded, cancer cell membrane‐coated nanoparticles	Tumor‐associated antigens, including MART1, TRP2, and gp100	CpG	N/A	B16F10
5[44]	Bacterial pathogen‐mimic vaccine backbones	OVA	CpG	N/A	E.G7‐OVA
6[45]	3D nanofibrous hydrogel	OVA	N/A	*α*PD‐1	E.G7‐OVA
7[41b]	PLGA‐R837@Cat nanoparticles	Tumor‐associated antigens during radiotherapy	R837	*α*CTLA‐4	CT26

### 3.1 Tumor Antigen Components

To elicit an effective antitumor response, cancer antigens must be taken up by DCs and cross‐presented for CD8^+^ T‐cell priming.^[^ ^46^ ^]^ Tumor antigens are essential components that can be used as cargo incorporated into biomaterials to enhance the efficacy of DC‐based vaccines. However, the exact tumor antigen components that induce immune responses have not completely been revealed, which limits the antigenic loading for iDCs to a certain extent. Multiple antigens have been used in experimental research to explore the efficacies of DC‐mediated immunotherapy. The most commonly used antigens are the model antigen protein ovalbumin (OVA) and the peptide SIINFEKL.^[^ ^47^ ^]^ These antigens have a defined peptide and corresponding tumor cell lines stably expressing the peptide, such as the thymoma cell line E.G7‐OVA.^[^ ^48^ ^]^ Moreover, some established tumor antigens, such as tissue differentiation antigens (such as gp100, and Melan‐A/MART‐1), have been proven to be specific melanoma antigens.^[^ ^49^ ^]^ For most tumors, the antigens that specifically activate DCs are unknown. In this condition, tumor cell lysates can serve as a compromise for DC‐mediated immunotherapy. However, the immunogenicity of tumor lysates is too low to evoke an adequate immune response. To address this inadequate immunogenicity, tumor‐associated antigens (TAAs) have been studied. For example, the autophagosome‐enriched vaccine (DRibbles), comprising a group of TAAs with short‐lived proteins (SLiPs) and defective ribosomal products (DRiPs), has been shown to have greater antitumor efficacy than tumor lysate vaccines.^[^ ^16^ ^]^ Cancer‐testis antigens (CTAs), which are aberrantly expressed in a wide array of advanced tumors, offer potential clinical biomarkers for immunotherapeutics in various malignancies.^[^ ^50^ ^]^ Oncofetal antigens, which are expressed on hematologic cancer cells but not in healthy cells, have been studied as specific antigens for DCs.^[^ ^51^ ^]^ In addition, immunogenic cell death (ICD) of tumor cells can be induced via various treatment methods. ICD‐released TAAs and damage‐associated molecular patterns (DAMPs), can act as endogenous antigens, which are engulfed by DCs and then presented to T cells.^[^ ^52^ ^]^ Inducing tumor pyroptosis can also release of TAAs to promote cascade‐amplification of the antitumor DC‐immune response.^[^ ^53^ ^]^ There have been significant advances in the identification of tumor antigens in recent years. Clinically effective CTL responses have been found to be induced by neoantigens derived from tumor‐specific mutations that accumulate in cancer. Preparing DC‐based vaccines using tumor‐specific neoantigens is a fascinating strategy for the manipulation of neoantigen‐specific T‐cell responses.^[^ ^54^ ^]^ Compared to TAAs, neoantigens have a higher specificity for tumor cells, thus inducing a more robust CTL response.^[^ ^55^ ^]^ With the development of sequencing technology, RNA sequencing technology can be used to identify somatic mutations in tumor cells, and the sequencing results can be used to make patient‐ and tumor‐specific neoantigens for DC‐based vaccines.^[^ ^56^ ^]^

The loading form of antigens is also an important factor in the activation of DCs. DCs loaded with free cancer antigens, for example, in the form of proteins, lysates, peptides, nucleic acids, and polysaccharides from autologous or allogeneic tumor cells, can be utilized in the preparation of traditional DC‐based vaccines.^[^ ^57^ ^]^ However, the delivery efficacy of these free antigens is too low, which leads to weak immune responses. Biomaterials can enhance antigen uptake by DCs, promoting localized antigen concentrations. Several properties of antigens, such as size, shape, surface charge, hydrophobicity, hydrophilicity, and receptor interactions, affect antigenic uptake by DCs. Antigens combined with biomaterials can induce antitumor immune responses that are stronger than those induced by free antigens. This optimization will result in a lower antigen dose than using free equivalents to elicit adequate immune responses.^[^ ^58^ ^]^ The reason is that the antigens loaded in biomaterials efficiently participate in the MHC‐I pathway and are more easily processed through the cross‐presentation pathway than those in a free‐protein state.^[^ ^59^ ^]^ In addition, some responsive biomaterials can not only directly kill tumor cells but also produce tumor‐related antigens from tumor cell residues in situ and deliver antigens to DCs for promoting the antitumor immune response.^[^ ^60^ ^]^ Therefore, the application of biomaterials for antigen delivery is promising in DC‐mediated cancer immunotherapy.

### 3.2 Immunostimulators for DC Maturation

Immunostimulatory molecules, particularly Toll‐like receptor (TLR) agonists, affect DC maturation. This effect is induced through myeloid differentiation factor 88 (MyD88)‐dependent TLR signals that drive I*κ*B‐kinase (IKK)2‐mediated phosphorylation of phagosome‐associated SNAP23. Phospho‐SNAP23 stabilizes SNARE complexes thereby orchestrating endosomal recycling compartment (ERC)‐phagosome fusion and leading to the accumulation of phagosomes with ERC‐derived MHC‐I. This cascade of reactions promotes the antigenic cross‐presentation of DCs.^[^ ^61^ ^]^ TLR agonists such as poly (I:C) (TLR3), LPS (TLR4), R848 or R837 (TLR7), and CpG (TLR9), promote the maturation of DCs, upregulate the expression of costimulatory molecules on the surface of DCs, and enhance the production of cytokines and chemokines.^[^ ^62^ ^]^

Synthetic biomaterials are capable of boosting antigenic presentation and DC activation when they are packaged with antigen and TLR agonists. Alloatti et al. documented that TLR4 engagement can induce Rab34‐dependent redistribution of lysosomal components and facilitate cross‐presentation by transiently delaying antigenic degradation.^[^ ^63^ ^]^ Additionally, CpG‐conjugated biomaterials were found to significantly upregulate costimulatory molecules and enhance cytokine production in splenic DCs.^[^ ^64^ ^]^ Jewell et al. demonstrated that poly (I:C) loaded with poly(lactic‐co‐glycolic acid) (PLGA) microspheres substantially amplifies DC activation in dLNs.^[^ ^65^ ^]^ Intranodally injected poly (I:C)‐loaded biomaterial mediated sustained poly (I:C) release in LNs, prolonging DC activation and robust CD8^+^ T‐cell priming compared with soluble adjuvant. Knockdown of the immune‐suppressor gene of DCs, signal transducer and activator of transcription‐3 (STAT3), induced cytotoxic T lymphocyte (CTL) responses that efficiently inhibited tumor growth.^[^ ^66^ ^]^ Heo et al. embedded R837 and siRNA in PLGA to activate DCs by the direct route and knockdown of STAT3. In addition to TLR agonists, the tumor necrosis factor (TNF) receptor family member OX‐40 has also been shown to have a critical costimulatory effect on DCs.^[^ ^67^ ^]^ The ligand of OX40 (CD252) is expressed on activated APCs, specifically on DCs, and regulating OX40 signaling strongly promotes the bioactivity of CD4^+^ and CD8^+^ T cells and counteracts Treg functions.^[^ ^68^ ^]^ Therefore, OX‐40 agents could serve as potential immunostimulators that boost the functions of DCs. In addition to OX‐40, glucocorticoid‐induced tumor necrosis factor receptor‐related protein (GITR) and tumor necrosis factor receptor superfamily 9 (4‐1BB) are promising targets for DC‐mediated immunotherapy. GITR agonists can promote effector T‐cell functions and inhibit Treg suppression. 4‐1BB signaling activation delivers a dual mitogenic signal for T‐cell activation and proliferation.^[^ ^69^ ^]^ Overall, the combinations of immunostimulators and synthetic biomaterials can enhance the activation of the DC‐induced innate immune system and thereby boost CTL responses.

### 3.3 Immune Checkpoint Blockade Agents

The essential purpose of DC vaccination is to stimulate antigen‐specific T cells to specifically recognize and kill cancer cells. Although DC‐based vaccines can activate CTL responses through the cross‐presentation pathway, the efficacies are hampered by different tumor escape mechanisms, which include reductions of T‐cell functions and viability in the immunosuppressive TME.^[^ ^70^ ^]^ In addition, the immune camouflage of tumor antigens, the downregulation of MHC‐I expression, the secretion of immunosuppressive cytokines, and the expansion and recruitment of negative regulatory pathways associated with Tregs and MDSCs can induce tumor escape.^[^ ^71^ ^]^ For example, CTLA‐4 is an intracellular protein in resting T cells, and when TCR engagement and a costimulatory signal occur through CD28, CTLA‐4 translocates to the cell surface. When it outcompetes CD28 for binding to costimulatory molecules of DCs (CD80/CD86) and provides a negative signal that inhibits T‐cell expansion and activation.^[^ ^72^ ^]^ The PD‐1 receptor has emerged as a dominant negative regulator of antitumor T‐cell effector function when engaged by its ligand PD‐L1, which is expressed on the surface of cells, such as TAMs, tumor‐associated DCs, Tregs, and fibroblasts within a tumor.^[^ ^73^ ^]^ PD‐1 is an immune checkpoint that produces inhibitory function via the tyrosine phosphatase SHP‐2, which dephosphorylates signaling molecules downstream of the TCR.^[^ ^74^ ^]^ High expression levels of PD‐L1 in the TME result in PD‐1‐mediated T‐cell exhaustion, inhibiting the antitumor CTL response.

Therapeutic DC vaccine‐induced antigen‐specific T‐cell responses are commonly observed; however, the clinical response rate is relatively poor. There is evidence that immune checkpoints hinder DC function, even resulting in the immune escape of tumors.^[^ ^75^ ^]^ The tumor escape dilemma can be overcome by using ICBs, thereby interrupting tumor growth.^[^ ^76^ ^]^ Recently, the application of ICBs has emerged as a critical antitumor strategy against multiple cancer types, such as melanoma, non‐small cell lung cancer, and renal‐cell carcinoma.^[^ ^39^, ^77^ ^]^ Antibodies against CTLA‐4/PD‐1 are the most commonly used ICBs. PD‐1 antibodies can rescue dysfunctional and exhausted CD8^+^ T cells. They both increase the tumor infiltration of effector T cell and synergistically decrease the levels of Tregs and MDSCs. Combination immunotherapies based on biomaterials and checkpoint inhibitor antibodies can increase the efficacy of DC‐based vaccines. For example, PD‐1 and CTLA‐4 antibodies have been used in combination with biomaterials to induce synergistic effects in DC‐mediated immunotherapy, which potently inhibited tumor growth.^[^ ^41^, ^45^, ^78^ ^]^ In addition, PD‐L1 molecue has been shown to bind the costimulatory molecule CD80 (B71) expressed on T cells and then deliver an inhibitory signal.^[^ ^79^ ^]^ In particular, PD‐L1 molecue expression on DCs was found to be higher than that on other cell types (CD3^+^CD8^+^ T cells, CD3^+^CD4^+^ T cells, CD19^+^ B cells, CD11b^+^F4/80^+^ macrophages, CD11b^+^Gr‐1^+^ MDSCs, and CD45^−^ tumor cells) in the TME, which suggests that blockade of PD‐L1 on DCs is essential for CTL activation.^[^ ^28b^ ^]^ Given the immune escape inhibitory effects of ICBs, they can be used to help biomaterial‐augmented DC vaccines overcome immunosuppression in the TME.

## 4 Rational Design of Biomaterials to Activate DCs

Apart from the effects of tumor antigens, immunostimulators, or ICBs, the development of rational biomaterial platforms is important to improve DC‐based immunotherapeutic outcomes.^[^ ^80^ ^]^ Biomaterial platforms, such as nanoparticles, microparticles, microneedles, and hydrogel scaffolds, can be engineered to spatially and temporally control the interactions of immune components with DCs.^[^ ^81^ ^]^ Tailoring the properties of biomaterials has recently emerged as an important strategy for the design of immune adjuvants. Numerous studies have sought to illustrate the physicochemical properties of biomaterials, such as targeting ligands,^[^ ^82^ ^]^ size,^[^ ^83^ ^]^ morphology,^[^ ^84^ ^]^ hydrophobicity,^[^ ^85^ ^]^ and surface charge,^[^ ^85^, ^86^ ^]^ in the regulation of the tumor vaccination cascade.^[^ ^87^ ^]^ As a whole, rational modification and design of biomaterials can augment antitumor immunotherapy by improving DC activation, creating local antigen‐rich niches, targeting dLN delivery, and controlling the time frame of vaccine delivery.

**Figure 4:**
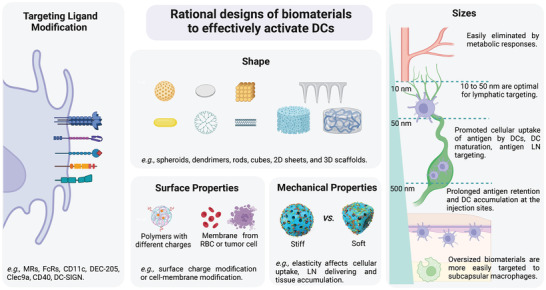
Rational design of biomaterials to activate DCs. Tailoring the properties of biomaterials is an important strategy for the design of immune adjuvants. Current studies illustrate that the properties of biomaterials, such as targeting ligands, size, morphology, surface charge and mechanical properties, need to be considered in the regulation of the DC‐mediated antitumor vaccination cascades. Rational modification and design of biomaterials can improve DC activation.

The immunostimulatory activities of biomaterials have been attributed to diverse mechanisms, such as the effective delivery of antigens, the “depot effect” for antigens, TLR‐independent signal transduction, antigen presentation, and release of immunomodulatory molecules.^[^ ^88^ ^]^ Delivery systems not only improve the stability and bioavailability of antigens but also protect a variety of antigens and costimulatory molecules from biodegradation. Biomaterials can be used as delivery systems, and antigens can be conjugated with or encapsulated in biomaterials.^[^ ^11b^ ^]^ In addition to the ability of biomaterials to deliver antigens and costimulatory molecules, their capacities for targeting, cellular uptake, and binding active molecules and their biosafety are important design parameters. When imaging biomaterials associated with DCs by specific receptors, the migration of DCs can be tracked.^[^ ^89^ ^]^ Biomaterial carriers can be modified with ligands for targeted site‐specific antigen delivery to DCs.^[^ ^90^ ^]^ In addition, the form of biomaterials can also improve antigen recognition, uptake, and processing by DCs by increasing endocytosis by tuning their size, shape, surface properties or mechanical properties. These factors affect cellular uptake, biocompatibility, blood circulation, tumor penetration, and lymphatic uptake and trafficking.^[^ ^91^ ^]^ Moreover, some emerging biomaterials, such as microneedles, hydrogels, and self‐assembled scaffolds, can be designed to create a local depot to improve innate immune cell infiltration, resulting in increased antigen uptake by DCs. Therefore, designing biomaterials with rational parameters is important for DC‐mediated immunotherapy (**Figure** **4**).

### 4.1 Targeting Ligand Modification of Biomaterials

The efficiency of antigen targeting to DCs depends on not only the choice of the delivery system but also the receptor‐ligand interactions.^[^ ^92^ ^]^ The administration of biomaterials with specific binding and target molecules can deliver antigens to targeted DCs and stimulate their maturation and activation.^[^ ^93^ ^]^ Various receptor molecules on DCs can be used as targeting sites. The receptors include mannose receptors (MRs), Fc receptors (FcRs), CD11c receptors, DEC‐205 receptors, C‐type lectin domain family member A (Clec9a), CD40 receptors, and DC‐specific intracellular adhesion molecule‐3 grabbing nonintegrin (DC‐SIGN). **Table** **2** summarizes biomaterials modified with different DC targeting ligands.

**Table 2:** Summary of biomaterials modified with different DC targeting ligands (Abbreviations: Man‐CTS‐TCL NPs, mannose‐modified chitosan nanoparticles loaded with whole tumor cell lysates; MAN‐ALG/ALG = OVA NPs, mannose‐functionalized alginate nanoparticle‐conjugated OVA; MAN‐OVA‐IMNPs, mannose‐modified lipid‐polymer hybrid nanoparticles OVA; MAN‐IMO‐PS, mannose‐functionalized lipid‐hybrid polymersomes; PMV, plasma membrane vesicles; pSiNP, porous silicon nanoparticles; CMVs, tumor cell membrane vesicles; N/A, not applicable)

No. ^[Ref.]^	Receptor	Ligands	Biomaterials	Modulation of DCs	Tumor Model
1[82a]	MR	Mannose	Man‐CTS‐TCL NPs	Promoted DC maturation, antigen uptake, and presentation	B16
2[94]	MR	Mannose	MAN‐ALG/ALG‐OVA NPs	Enhanced DC antigen uptake, cytosolic release, maturation, and antigen cross‐presentation	E.G7‐OVA
3[95]	MR	Mannose	MAN‐OVA‐IMNPs	Enhanced cellular uptake, cytokine production, and maturation of DCs.	E.G7‐OVA
4[96]	MR	Mannose	MAN‐IMO‐PS	Promoted efficiently internalized by DCs, and enhanced cross‐presentation and cytokine production	E.G7‐OVA
5[97]	FcRs	IgG Fc fragment	liposomes or AuNPs conjugated to the Fc fragment	Promoted Antigen uptake and DC immunological response	N/A
6[98]	DEC‐205	Anti‐CD205 mAbs	Polymeric NPs	Increased receptor‐mediated uptake of nanovaccine by DCs and DC migration to dLNs	N/A
7[99]	DEC‐205	Anti‐DEC‐205 mAb	PLGA NPs	Increased the amount of IL‐10, produced by DCs and T cells	N/A
8[100]	CD11c	Anti‐CD11c mAbs	liposomes‐coated AuNCs	Promoted uptaken by iDCs and DC migration	B16‐F10
9[101]	CD11c and DEC‐205	scFv	liposomes/PMV	Promoted targeting of antigen to DCs	B16‐OVA
10[102]	CD40	Anti‐CD40 mAbs	PLGA NPs	Led to very efficient and selective delivery to DC	B16‐OVA
11[82c]	CD40, DEC‐205, or CD11c	Anti‐CD40, anti‐DEC‐205, or anti‐CD11c mAbs	PLGA complex	More efficiently targeted to and internalized by DC	N/A
12[103]	DC‐SIGN	chol‐Apt	CMVs complex	Specific targeting of CMVs to DCs	CT26
13[104]	CD11c/DC‐SIGN	CD11c mAbs/DC‐SIGN mAbs	CD11c‐pSiNP/DC‐SIGN‐pSiNP	Enhanced cellular uptake of DCs	N/A
14[105]	Clec9a	Anti‐Clec9A mAb	Antigen‐Clec9A‐TNE	Targeted and activated cross‐presenting DCs	B16‐F10
15[106]	SR‐B1	*α*‐peptide	*α*‐Ap‐FNP	Rapid dLN accumulation and targeting of DCs in dLN	E.G7‐OVA

**Figure 5:**
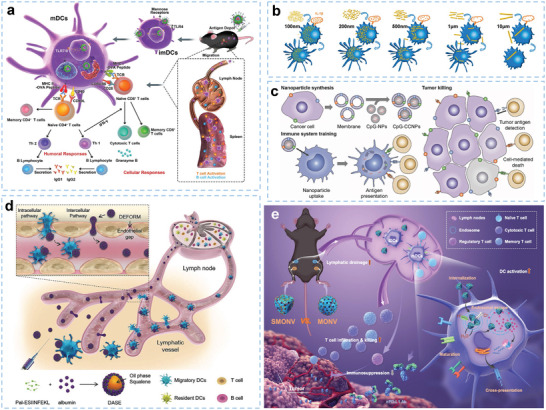
Representative design of biomaterials with different parameters for DC‐mediated immunotherapy. a) Illustration of mannose‐modified nanovaccines for DC‐mediated cancer immunotherapy. Reproduced with permission.^[^ ^95^ ^]^ Copyright 2019, American Chemical Society. b) Size design strategies for DC‐targeted delivery systems. Reproduced with permission.^[^ ^112^ ^]^ Copyright 2019, American Chemical Society. c) Surface modified biomaterials for DC‐based antitumor vaccination. Reproduced with permission.^[^ ^43^ ^]^ Copyright 2017, Wiley‐VCH. d) Schematic illustration of the deformable strategy of LN transfer by designed softer biomaterials. Reproduced with permission.^[^ ^113^ ^]^ Copyright 2021 Wiley‐VCH. e) Schematic illustration showing the underlying mechanism by which SMONV enhances DC‐mediated antitumor immune responses. Reproduced with permission.^[^ ^114^ ^]^ Copyright 2022, Wiley‐VCH

MRs are C‐type lectin receptors that are highly expressed on the surface of DCs.^[^ ^107^ ^]^ White et al. designed mannose‐containing ligands to modify liposomes to increase antigenic uptake by DCs. However, the expression levels of CD86 and CD40 on the surfaces of DCs were not enhanced, which meant that mannosylated liposomes did not enhance DC maturation.^[^ ^108^ ^]^ To refine conventional mannosylated biomaterials, Ishii et al. designed oligomannose‐coated liposomes for preferential uptake by DCs, which promoted the DC maturation, activation, and trafficking into lymphoid organs and induced strong antigen‐specific T‐cell immunity.^[^ ^109^ ^]^ Mannosylated dendrimer antigens with stronger DC binding avidity have also been exploited to enhance the antigenic presentation and maturation of DCs.^[^ ^110^ ^]^ In addition to liposomes and dendrimers, other nanoparticles have been modified with mannose ligands to target and stimulate DC activation. Xu et al. documented that mannose‐modified lipid‐calcium‐phosphate nanoparticles enhanced and prolonged OVA antigen deposition in dLNs and ensured persistent antigen loading and DC stimulation.^[^ ^111^ ^]^ Shi et al. documented that chitosan nanoparticles modified with mannose moieties can specifically target DCs and promote IFN‐*γ* secretion by CTLs.^[^ ^82a^ ^]^ Zhang et al. constructed novel versatile and mannose‐targeting nanovaccines for the codelivery of OVA antigen and TLR agonists.^[^ ^95^ ^]^ The MAN‐OVA‐IMNPs can be efficiently internalized by iDCs via mannose‐targeting to MR and mannose decoration, thus enhancing dLN targeting and promoting the induction of antigen‐specific T cells (**Figure** **5**a).

In addition to MR receptors, FcRs as endocytic receptors can be used as target receptors. The FcR‐associated chain is closely related to DC maturation and antigen presentation. The Fc fragment can be conjugated to liposomes or gold nanoparticles (AuNPs) to target FcRs of human DCs, thereby facilitating antigen delivery and inducing superior antigen presentation.^[^ ^97^ ^]^ Kawamura et al. designed an IgG‐modified OVA‐containing liposome for targeting FcR on DCs.^[^ ^115^ ^]^ Mice immunized with activated DCs efficiently prevented the growth of lymphoma cells that expressed OVA. CD11c and DEC‐205 receptors are expressed exclusively on DCs and participate in the antigen capture and presentation process. Single‐chain full‐length variable antibody fragments (scFv) modified on plasma membrane vesicle surfaces can target DEC‐205 and CD11c on DCs, and stimulate strong spleen‐specific CTL responses that protect against B16 tumor growth.^[^ ^101^ ^]^ CD40 is a TNF‐*α* family receptor expressed on DCs, and agonistic CD40 ligation on mDCs is also critical in triggering adequate T‐cell‐mediated immune responses.^[^ ^116^ ^]^ DCs can be activated by targeting CD40 using specific ligands. In a study, DC‐surface molecules, including CD40, DEC‐205, and CD11c, were targeted by PLGA nanoparticles coupled with specific monoclonal antibodies.^[^ ^82c^ ^]^ It was found that internalization of CD40‐, DEC‐205‐ or CD11c‐targeted nanoparticles stimulated both IL‐12 secretion and the expression of costimulatory molecules. Compared to nontargeted nanoparticles, targeted nanoparticles are efficient in stimulating DC‐mediated CTL responses. DC‐SIGN (CD209) is a DC‐specific C‐type lectin‐like cell‐surface receptor. Biomaterials with antibodies that recognize DC‐SIGN can enhance its uptake by DCs.^[^ ^117^ ^]^ Stead et al. developed porous Si nanoparticles displaying DC‐SIGN and CD11c monoclonal antibodies, which were easily taken up by splenic and peripheral blood DCs.^[^ ^104^ ^]^ In addition, Clec9a is selectively expressed on mouse CD8*α* ^+^ DCs. Activated Clec9a signaling promotes cross‐presentation, and elicits robust CTL responses.^[^ ^118^ ^]^ Zeng et al. encapsulated an antigen in a Clec9A‐targeting nanoemulsion that enabled escape of nonspecific phagocytosis and targeting of antigens to CD8*α* ^+^ DCs.^[^ ^105^ ^]^ In summary, DC‐specific ligands fused in biomaterials can target DC receptors and increase the efficiency of antigenic delivery.

### 4.2 Sizes and Shapes of Biomaterials

The size of particulate biomaterial carriers greatly influences biological effects such as the lymphatic uptake of delivery vehicles and lymphatic trafficking of biomaterials. Biomaterials with sizes ranging from 10 to 50 nm are optimal for lymphatic targeting and can leave the interstitial space of healthy tissues through lymphatic drainage. Sai et al. showed that 25 nm nanoparticles transported by interstitial flow could target half of the DCs residing in dLNs, while only 10% of 100 nm biomaterials were efficient after intradermal injection.^[^ ^83a^ ^]^ It has also been documented that 20 nm biomaterials are more readily transported into the lymphatics than 100 nm biomaterials. In addition, 20 nm biomaterials were internalized by nearly half of the lymph node DCs without targeting ligands, while only 6% of 100 nm biomaterials were internalized.^[^ ^119^ ^]^ Although the rate of diffusion of smaller biomaterials is greater than the convective velocity, biomaterials that are too small (<20 nm) do not maintain a long‐lasting effect in lymphatics because they are easily eliminated by metabolic responses. Conversely, larger biomaterials have more difficulty entering the dLNs and are not easily lost due to lymph node retention. Thus, the probability of biomaterials remaining in the dLNs is positively correlated with their size. Although increasing the sizes of biomaterials can enhance their retention inside the lymphatics, oversized biomaterials are more easily targeted by subcapsular macrophages, reducing their uptake by DCs.^[^ ^120^ ^]^ Furthermore, biomaterial sizes significantly affect the efficiency of antigenic uptake by DCs and the subsequent maturation of DCs.^[^ ^112^ ^]^ Hence, more detailed studies aimed at optimizing the sizes of biomaterials to enhance the permeability and retention (EPR) effect and maximize activated DC‐mediated immune responses are needed. In addition, the size of the biomaterials determines the cellular uptake of antigens and immunostimulatory molecules by DCs. Wang et al. designed rod‐shaped hydroxyapatite particles with lengths of ≈100 nm, 200 nm, 500 nm, 1 µm, and 10 µm to clarify the underlying mechanism accounting for the size‐dependent effect of biomaterials on DC‐mediated antitumor immune responses (Figure 5b).^[^ ^112^ ^]^ Rod‐shaped hydroxyapatite particles with shorter lengths promoted cellular uptake of antigen by DCs, DC maturation, and antigen dLN targeting; and the rods with longer lengths prolonged antigen retention and DC accumulation at the injection sites. This study provides a reference architecture for the future size design of DC‐targeted delivery systems, including an in‐depth understanding of antigen delivery and immune activation mechanisms in a size‐dependent manner.

In addition to size, biomaterial shape is also an important parameter affecting biodistribution, cellular uptake, and toxicity. Biomaterials with various shapes, such as spheroids, dendrimers, rods, cubes, 2D sheets, and 3D scaffolds, confer different functions (surface attachment, encapsulation, or biomaterial labeling, etc.) for the simultaneous or sequential target‐specific delivery of multiple antigens or costimulatory molecules to DCs.^[^ ^121^ ^]^ Niikura et al. documented that different shapes of AuNPs elicited robust immune responses through different cytokine pathways. The researchers found that spherical and cubic AuNPs significantly induced IL‐6, IL‐12, TNF‐*α*, and GM‐CSF expression, while only rod‐shaped AuNPs activated IL‐18 and IL‐1*β* secretion via a DC inflammasome‐dependent pathway.^[^ ^122^ ^]^ 2D nanomaterials possess outstanding properties, such as remarkable light‐weight and high surface‐to‐volume ratio. Graphene oxide (GO) nanosheets can be designed as a multifunctional vaccine platform for neoantigen‐based DC vaccines. Xu et al. reported that graphene oxide sheets coated with polymers, when administered through the intradermal route, promoted the maturation of DCs and evoked a stronger immune response than clinically used aluminum nanoparticles.^[^ ^123^ ^]^ Xu and co‐workers developed a reduced graphene oxide nanosheet (RGO)‐PEG carrying CpG neoantigen as a multifunctional nanovaccine platform. RGO‐PEG drastically improves vaccine delivery to dLNs after subcutaneous vaccination and then induces intracellular reactive oxygen species (ROS) in DCs, guiding antigen processing and presentation to T cells.^[^ ^124^ ^]^ Furthermore, RGO‐PEG triggers intracellular ROS generation in DCs, resulting in alkalization of endolysosomes and strong and sustained antigen presentation. The strategy elicits potent and durable antigen‐specific CTL responses and suppresses tumors. Recently, biomaterials with unique shapes, such as microneedles or 3D scaffolds, have been designed and studied for DC activation. Bioscaffolds locally recruit and program host DCs to induce effective innate and adaptive immune responses. Dellacherie et al. reported mesoporous silica microrod‐based scaffolds to generate high antibody titers against synthetic peptides and other small antigens. In this work, subcutaneously injected microrods spontaneously assembled into 3D scaffolds and consequently recruited and activated DCs.^[^ ^125^ ^]^ This specific shape was also proven to have unique advantages for activating DCs. As such, shape is a crucial characteristic of biomaterials that should be given more attention.

### 4.3 Surface Properties of Biomaterials

Surface properties affect biomaterial internalization by DCs.^[^ ^11a^ ^]^ Biomaterial carriers with cationic surfaces are prone to adhere to cell membranes and are therefore more easily internalized by DCs than neutral or anionic biomaterials. This notion is attributed to the fact that cell membranes possess negative charges and have a greater affinity for positively charged molecules.^[^ ^126^ ^]^ A positive charge on biomaterials also promotes the maturation of DCs.^[^ ^127^ ^]^ For example, positively charged polyethyleneimine (PEI)‐modified mesoporous silica microrods incorporated with neoantigens and immunostimulators (CpG and GM‐CSF) induced an eightfold increase in the number of neoantigen‐specific peripheral CD8^+^ T cells.^[^ ^40^ ^]^ Previous studies demonstrated that cationic aluminum hydroxide nanoparticles can enhance OVA uptake by DCs, and sequentially activate DCs and B3Z T cells in culture.^[^ ^59a^ ^]^ Yue et al. found that positively charged modified biomaterials can escape lysosomes after internalization, while negatively and neutrally charged nanoparticles mainly remain inside lysosomes.^[^ ^128^ ^]^ Superparamagnetic iron oxide nanoparticles with anionic coatings exhibited superior adjuvant potential for the enhancement of OVA cross‐presentation and T‐cell activation. This effect is attributed to the secretion of IL‐1*β*.^[^ ^89a^ ^]^ Fytianos et al. demonstrated that cationic charges on the AuNP surface induced higher antigen uptake by human monocyte‐derived DCs.^[^ ^129^ ^]^ However, notably, Verma et al. established that cationic nanoparticles, when passed through cell membranes, generate transient holes and result in potential cytotoxicity.^[^ ^130^ ^]^ In addition to charge modification, membranes derived from cells, such as erythrocytes (red blood cells, RBCs) or tumor cells, can be used to modify antigen delivery platforms. RBC membranes can prevent antigen clearance during blood circulation and effectively deliver antigens to targeted DCs.^[^ ^131^ ^]^ Biomaterials modified with tumor cell‐derived components can also exhibit potent multiantigenic immune responses (Figure 5c).^[^ ^43^ ^]^ An increasing number of novel surface modification methods will lead to the functional improvement of biomaterials in the future.

### 4.4 Mechanical Properties of Biomaterials

Recently, biomaterial elasticity has been proven to play a pivotal role in the nano‐biointerface, such as cellular uptake,^[^ ^132^ ^]^ circulation,^[^ ^132^, ^133^ ^]^ and tissue accumulation.^[^ ^132^, ^134^ ^]^ How the mechanical properties of biomaterials influence the functions of DCs remains largely unclear. Song et al. engineered deformable albumin‐stabilized emulsions (DASE) for lymph‐node vaccine delivery (Figure 5d).^[^ ^113^ ^]^ The softer DASE (≈330 nm) can attach to and deform between DCs and adjust their sizes to pass through the endothelial gaps (20–100 nm), which contributes to the self‐adaptive deformability of DASE and leads to direct LN transfer (intercellular pathway). Compared with stiff particles, DASE‐based nanovaccines improve antigen accumulation and LN drainage and potently evoke cellular immune responses, thus increasing the survival rate of tumor‐bearing mice. Moreover, Li et al. reported a soft mesoporous organosilica‐based nanovaccine (SMONV) and demonstrated that the elastic nanovaccine SMONV generates a robust antitumor immune response (Figure 5e).^[^ ^114^ ^]^ Mechanistically, SMONV achieves efficient cytosolic delivery of antigens to DCs via enhanced elasticity‐dependent cellular uptake and endosomal escape, leading to increased antigen cross‐presentation while simultaneously activating DC maturation with high efficiency. Meanwhile, the elastic nanovaccine elevates lymphatic drainage of antigens in vivo, thus stimulating potent humoral and cellular immunity. Impressively, elastic SMONV effectively inhibits tumor growth by evoking antigen‐specific CD8^+^ T‐cell immune responses, mitigating Treg cell‐mediated immunosuppression, and increasing memory T‐cell populations. These findings innovatively highlight the importance of elasticity in rationally designing nanovaccines and suggest that the prepared SMONV offers a facile and effective strategy for DC‐mediated tumor immunotherapy.

## 5 Engineered Biomaterials as Delivery Platforms for Activating DCs

**Figure 6:**
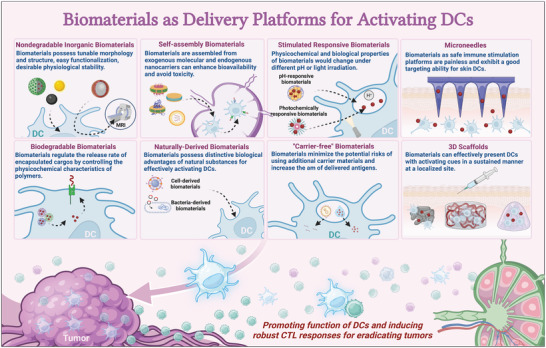
Biomaterials as delivery platforms for activating DCs. Biomaterial strategies can be based on various materials and cargo encapsulation/delivery mechanisms. For example, nondegradable inorganic biomaterials possess tunable morphology and structure, easy functionalization, and desirable physiological stability. Biodegradable biomaterials regulate the release rate of encapsulated cargos by controlling the physicochemical characteristics of polymers. Self‐assembly biomaterials assembled from exogenous molecular and endogenous nanocarriers can enhance bioavailability and avoid toxicity. Naturally derived biomaterials possess distinctive biological advantages of natural substances for effectively activating DCs. The physicochemical and biological properties of stimulated responsive biomaterials change under different pH values or light irradiation. “Carrier‐free” biomaterials minimize the potential risks of using additional carrier materials and increase the amount of delivered antigens. Microneedles, as safe immune stimulation platforms, are painless and exhibit a good targeting ability for skin DCs. 3D scaffolds can effectively present DCs with activating cues in a sustained manner at a localized site.

In the past few decades, studies have aimed to improve the efficacy of DC‐based vaccines by developing biomaterial‐based delivery systems.^[^ ^135^ ^]^ Particulate biomaterials are widely used for antigen delivery.^[^ ^136^ ^]^ Their unique physiological properties improve the efficacy of DC functions. Nondegradable biocarriers are relatively stable and exhibit long‐lasting immune adjuvant abilities. Biodegradable biomaterials are designed and synthesized because their degradation in vivo can be controlled over a predetermined period of time. To improve biocompatibility, some self‐assembly biomaterials, such as lipoprotein nanodiscs and lipid carriers, have been applied to deliver antigenic molecules. Biogenic compositions possess immunological activities that can be used to modify biomaterials and enhance their adjuvant properties. Biomaterials can also be designed to accurately release antigens or immune‐active molecules in controlled‐release patterns. In addition, scholars have explored the “carrier‐free” antigen delivery system, which is made from antigens and adjuvants and can induce activation of DCs without using additional biocarriers. Moreover, microneedle biomaterials and 3D scaffolds have been proven to be excellent antigenic vectors for promoting DCs. The development of diverse delivery systems provides strong support for DC‐based cancer immunotherapy (**Figure** **6**).

### 5.1 Nondegradable Inorganic Biomaterials

#### 5.1.1 Inorganic Nano‐Biomaterials

**Figure 7:**
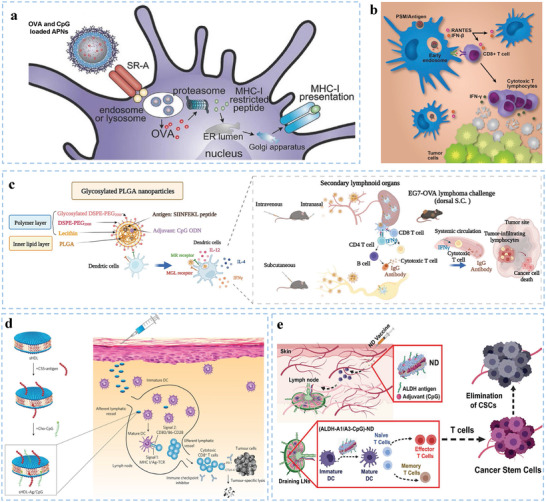
Representative biomaterials as delivery platforms for activating DCs. a) Nondegradable nano‐biomaterials enhance DC‐mediated immunity. Reproduced with permission.^[^ ^59a^ ^]^ Copyright 2017, Wiley‐VCH. b) Inorganic micro‐biomaterials (PSMs) loaded with liposomal antigens are efficiently internalized by DCs and trafficked to early endosomes for efficient cross‐presentation. Reproduced with permission.^[^ ^59b^ ^]^ Copyright 2015, Elsevier. c) The effect of glycosylated PLGA nanoparticle vaccines by enhancing DC targeting in the spleen and lymph nodes. Reproduced with permission.^[^ ^157^ ^]^ Copyright 2022, Elsevier B.V. d) Synthesized high‐density lipoprotein nanodiscs efficiently co‐deliver antigen and CpG to dLNs and promote the maturation and antigen presentation of DCs. Reproduced with permission.^[^ ^41a^ ^]^ Copyright 2016, Nature Publishing Group. e) Vaccination against cancer stem cells (CSCs) with nanodiscs (NDs) carrying ALDH epitopes for activating DCs. Reproduced with permission.^[^ ^160^ ^]^ Copyright 2020, American Chemical Society.

Inorganic nano‐biomaterials hold great promise due to their intrinsic characteristics, such as tunable morphology and nanostructure, easy functionalization, desirable physiological stability, and unique physiochemical properties (optical, electrical, acoustic, and magnetic properties).^[^ ^137^ ^]^ As the most common non‐degradable biomaterials, inorganic nanoparticles have been comprehensively studied in delivering tumor antigens to DCs. Among inorganic nanoparticles, aluminum‐based biomaterials have been used as immune adjuvants for the development of human vaccines.^[^ ^138^ ^]^ The *α*‐alumina nanoparticles conjugated to either OVA or DRibbles induced efficient autophagy‐dependent cross‐presentation, resulting in effective tumor regression.^[^ ^30b^ ^]^ The aluminum nanoparticles promoted antigenic cross‐presentation via an active proteasome‐dependent signaling pathway in DCs (**Figure** **7**a). Aluminum nanoparticles can also enhance the maturation of DCs by upregulating DC costimulatory molecules.^[^ ^59a^ ^]^ Various nanosized aluminum‐based nanoparticles, such as aluminum hydroxide nanoparticles, which serve as antigen carriers and/or adjuvants have been recently developed. These agents promote lysosomal escape, cytosolic delivery, and antigen cross‐presentation by DCs.^[^ ^42^, ^139^ ^]^ Sokolovska et al. reported that aluminum hydroxide nanoparticles promoted antigen presentation of DCs by enhancing the expression of maturation markers and cytokine secretion. These effects induced T helper cell differentiation, and this phenomenon was also observed using aluminum phosphate as an adjuvant.^[^ ^140^ ^]^ In addition to aluminum‐based nanoparticles, AuNPs have also been shown to have importance in DC nanovaccines. This type of nanoparticle is immunologically inert and nontoxic.^[^ ^141^ ^]^ Zhang et al. exploited stepwise electrostatic interactions between peptide antigens and TLR agonists to construct immune‐polyelectrolyte multilayers that self‐assembled on AuNPs. These modified AuNPs were then efficiently internalized by primary DCs and induced antigen‐specific T‐cell proliferation.^[^ ^141^ ^]^ Silica nanoparticles have also been shown to have good biocompatibility. Specifically, mesoporous silica nanoparticles have attracted increasing attention in DC‐mediated vaccine applications due to their high surface areas, internal pore volumes, and surface functionality.^[^ ^142^ ^]^ Inorganic metal biomaterials with bioimaging functions have also been used to label DCs. For instance, superparamagnetic iron oxide (SPIO) nanoparticles exert multifunctional roles in DC immunotherapy using their unique photothermal and magnetic resonance imaging (MRI) functions.^[^ ^143^ ^]^

In addition to single‐component inorganic nanoparticles, multifunctional composite nanoparticles have been designed to activate DCs. Ca, Mg, and Zn are essential elements in promoting antitumor immunity. Wang et al. demonstrated that mesoporous silica nanospheres doped with Ca, Mg, or Zn (MS–Ca, MS–Mg, and MS–Zn nanospheres, respectively) induce enhanced DC‐mediated stimulation of the CD8^+^ T‐cell population compared to pure mesoporous silica.^[^ ^144^ ^]^ Multifunctional core–shell nanoparticles have also been designed for DC‐based vaccines. The shell and core of these multifunctional core–shell nanoparticles consist of different materials that possess multiple properties and functions. Due to the magnetic effect of Fe_3_O_4_, multifunctional core–shell nanoparticles with Fe_3_O_4_ cores can be used in MRI. Iron oxide–zinc oxide core–shell nanoparticles can load carcinoembryonic antigens into DCs, enhance contrast for lymphoid tissues and provide high‐resolution DC images in vivo.^[^ ^145^ ^]^ These nondegradable inorganic nanoparticles exhibit promise in DC‐based vaccines.

#### 5.1.2 Inorganic Micro‐Biomaterials

Compared to nanoscale biomaterials, micro‐biomaterials have a larger surface area for a single particle to adsorb antigens. Various microparticles are candidates for DC‐based vaccine adjuvants. They can serve as antigen depots by carrying antigens, thereby protecting them from enzyme degradation and ultimately stimulating antigen presentation by DCs. Porous silicon microparticles were used as safe vehicles for the effective delivery of gene‐silencing agents in the past.^[^ ^146^ ^]^ Xia et al. studied discoid porous silicon microparticle DC‐based vaccine adjuvants (Figure 7b). The antigens inside the nanopores of porous silicon microparticles were effectively internalized by DCs and transported to early endosomes for efficient cross‐presentation.^[^ ^59b^ ^]^ Meanwhile, DC phagocytosis of porous silicon microparticles induced type I interferon responses that promoted DC functions and CTL clonal expansion. Zhu and coworkers utilized a mesoporous silicon microparticle as a multifunctional vector for delivering the TRP2 peptide and CpG to DCs.^[^ ^147^ ^]^ These micro‐biomaterials offer positive contributions to the development of antigen delivery systems for DC activation.

### 5.2 Biodegradable Biomaterials

Recently, polymer nanoparticles have been used as specialized biocompatible platforms for the delivery of antigens and immune molecules to DCs. For example, biodegradable polymers, such as poly(lactide‐co‐glycolide) (PLG),^[^ ^148^ ^]^ poly(methacrylic acid) (PMA(SH)),^[^ ^149^ ^]^ and PLGA,^[^ ^150^ ^]^ are commonly used polymer nanoparticles. These polymeric biodegradable biomaterials can regulate the release rate of encapsulated cargos by controlling the physicochemical characteristics of polymers.^[^ ^151^ ^]^ PLGA nanoparticles are the most common biodegradable polymer. They can evoke strong T‐cell responses even at low doses by loading antigens and adjuvants,^[^ ^152^ ^]^ which can minimize potential side effects. Shen et al. showed that antigen‐encapsulated PLGA nanoparticles could continuously release antigens when internalized in DCs. As such, these nanoparticles provided an intracellular store for persistent antigen presentation for several days.^[^ ^153^ ^]^ Silva et al. documented that vaccination in vivo with PLGA nanoparticles co‐encapsulating OVA and poly (I:C) not only significantly induced CD8^+^ T‐cell priming, but also resulted in a balanced Th1/Th2‐type antibody response.^[^ ^154^ ^]^ Tacken et al. showed that in comparison with administration of an equivalent free vaccine, codelivery of antigens and TLR ligands co‐encapsulated in PLGA nanoparticles showed 100‐fold improvement in eliciting antigen‐specific immunity, while effectively reducing serum cytokine levels.^[^ ^155^ ^]^ However, the disadvantages of PLGA nanoparticles such as low encapsulation efficiencies, limit their clinical applications.^[^ ^156^ ^]^ Hybrid lipid‐polymer nanoparticles possess the advantages of biodegradable polymeric nanoparticles and biomimetic liposomes. Chou et al. developed glycosylated PLGA nanoparticles loaded with tumor antigens and CpG for delivering cargos and enhancing the targeting ability to DCs in secondary lymphoid organs, thereby achieving efficient antitumor effects (Figure 7c).^[^ ^157^ ^]^ De Koker et al. described polyelectrolyte microcapsules (PMs) containing poly‐L‐arginine shells and CaCO_3_ cores as biodegradable microcarriers, which efficiently deliver antigens to promote antigen uptake by DCs, thus allowing DCs to efficiently process antigens into peptides and allowing superior presentation to both CD4^+^ and CD8^+^ T cells.^[^ ^158^ ^]^ Qiu et al. designed nanoplexes with antigenic peptides and poly‐(propylacrylic acid) (pPAA) to activate endosomal escape. These carriers evoked DC‐mediated strong CTL activation and prolonged the survival time of melanoma tumor‐bearing mice.^[^ ^159^ ^]^ Therefore, biodegradable nano‐biomaterials with excellent properties have tremendous potential to enhance the efficiency of DC‐based vaccine applications.

### 5.3 Self‐Assembly Biomaterials

Biomaterials assembled from exogenous molecular and endogenous nanocarriers would not only enhance bioavailability in dLNs, but also avoid potential toxicity. As an antigenic peptide carrier, high‐density lipoprotein (sHDL) nanodiscs consisting of phospholipids and apolipoprotein A1 (ApoA1)‐mimetic peptides can avert triggering adverse autoimmunity (Figure 7d). Using sHDL nanodiscs to adsorb CpG and tumor neoantigens, Kuai et al. prepared homogeneous, stable, and ultrasmall nanodiscs, which promote strong and durable DC antigen presentation.^[^ ^41a^ ^]^ In addition, nanodiscs evoke a 47‐fold greater frequency of neoantigen‐specific CTLs than soluble vaccines. Based on sHDL, Qian et al. designed an ultrasmall biocompatible tumor antigen peptide delivery platform that targeted mDCs localized within dLNs via the scavenger receptor class B1 pathway and enhanced antigenic peptide presentation of DCs.^[^ ^106^ ^]^ In addition, based on the features of synthetic high‐density lipoprotein nanodiscs and detoxifying intracellular aldehydes of aldehyde dehydrogenase (ALDH), Hassani Najafabadi et al. prepared ALDH nanodiscs to deliver ALDH epitope peptides to DCs and elicit T‐cell responses against ALDH^high^ cancer stem cells (CSCs). ALDH nanodiscs have attractive advantages, such as safety, good characterization, amenability for scalable manufacturing, mediating the codelivery of antigens and adjuvants to DCs in dLNs, and promoting antigen processing and presentation by DCs (Figure 7e).^[^ ^160^ ^]^ Albumin is a natural carrier with multiple, versatile, intrinsic binding sites for biomolecules, and drugs. Zhu et al. used albumin nanoparticles as an antigen peptide carriers for cancer immunotherapy. In the dLNs, these nanovaccines enhanced the exposure of antigenic peptides and CpG to DCs, thereby eliciting the generation of peripheral antigen‐specific CTLs with immune memory.^[161]^

In addition to peptides, mRNA with an attractive safety profile is considered to be a reliable form of tumor antigen.^[^ ^162^ ^]^ Lipid carriers have been extensively studied due to their high biosafety profiles, ease of manufacturing, and ease of quality control. It has been shown that lipid carriers can adjust the net charge of mRNA, which protects RNA from extracellular ribonucleases and mediates the efficient uptake and expression of the encoded antigen by DCs in various lymphoid compartments. Lipid carriers can be bioengineered to escape endosomes via the proton sponge effect. This effect protects mRNA from degradation by RNases and facilitates its uptake by DCs.^[^ ^163^ ^]^ DCs can be targeted precisely and effectively in vivo using intravenously administered RNA‐lipoplexes. RNA‐lipoplexes encoding neoantigens evoked strong effector T‐cell immune responses and induced rejection of progressive tumors in three melanoma patients in a phase I dose‐escalation trial.^[^ ^164^ ^]^ Self‐assembled biomaterials represent a widely applicable DC‐activated system that efficiently delivers antigens into LNs to activate DCs, ameliorate side effects, and induce potent and durable T‐cell responses.

### 5.4 Naturally Derived Biomaterials

#### 5.4.1 Cell‐Derived Biomaterials

Cell‐derived functional biomaterials have emerged as attractive therapeutic agents due to the distinctive biological advantages of natural substances, including long‐term circulation, tumor‐specific targeting, and immune modulation.^[^ ^165^ ^]^ Cell membranes have an important role in cellular targeting and cell‐to‐cell interactions. Enhancement of the bio‐interfacing properties of nanoparticles can be achieved directly by extracting and coating cell membranes on a nanoparticulate core.^[^ ^166^ ^]^ To enhance DC targeting and antigen‐presentation properties, Guo et al. designed erythrocyte membrane‐enveloped PLGA nanoparticles to load hgp100 and TLR‐4 agonists. The nanovaccines retained protein contents in outer erythrocyte membranes and increased DC uptake in vitro.^[^ ^131^ ^]^ Tumor cell‐derived biomaterials contain innate signals and tumor antigen profiles that endow them with vaccine ability. Yang et al. utilized a cancer cell membrane that had been modified by mannose to encapsulate nanoparticles and TLR‐7 agonists to develop cancer nanovaccines. This modification enhanced uptake by DCs and triggered effective antitumor immune responses.^[^ ^167^ ^]^ Kroll et al. coated B16‐F10 mouse melanoma cell membranes on PLGA nanoparticles that had been loaded with CpG. Cancer cell membrane‐coated nanoparticles (CpG‐CCNPs) were efficiently internalized by DCs, which suppressed tumor growth and enhanced mouse survival by combined with administration of CTLA‐4 and PD‐1 ICBs.^[^ ^43^ ^]^ Li et al. developed an engineered magnetosome that was wrapped with cancer cell membranes decorated with anti‐CD205.^[^ ^168^ ^]^ This magnetosome promoted antigenic recognition and uptake by DCs, thereby facilitating antigenic cross‐presentation.

**Figure 8:**
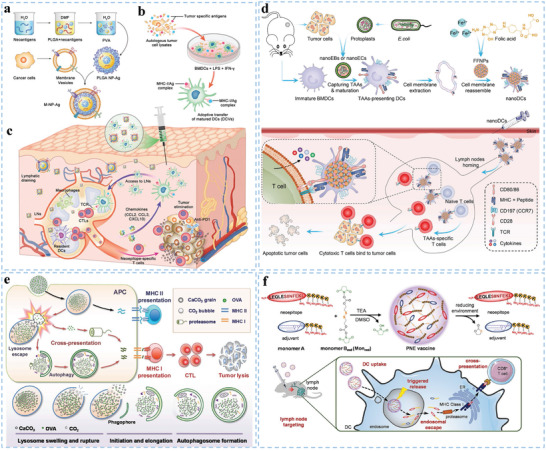
Representative naturally derived, stimulated responsive, and “Carrier‐free” biomaterials. Schematic illustration of cell‐derived nanovaccines, including a) biomaterial design, b) adaptive DC transfer, and c) their combinatorial immunization effects on personalized antitumor immunity. Reproduced with permission.^[^ ^171^ ^]^ Copyright 2021, Wiley‐VCH. d) Schematic illustration of bacteria‐derived personalized nanovaccines (nanoDCs) for promoting TAA presentation and DC‐mediated tumor immunotherapy. Reproduced with permission.^[^ ^175^ ^]^ Copyright 2022, Wiley‐VCH. e) pH‐responsive antigen‐doped CaCO_3_ nanoparticles, the schematic diagram showing antigen‐doped CaCO_3_ nanomissiles induced antigen cross‐presentation by lysosomal escape and autophagy. Reproduced with permission.^[^ ^176^ ^]^ Copyright 2018, Wiley‐VCH. f) The synthesis, antigen release, and DC uptake of “carrier‐free” biomaterials. Reproduced with permission.^[^ ^177^ ^]^ Copyright 2020, American Chemical Society.

The activation mechanism of DCs by tumor cell‐derived biomaterials has also been studied in recent years. It has been reported that tumor cell‐derived microparticles (T‐MP) activate lysosomal pathways after being endocytosed by DCs. T‐MP increases lysosomal pH through NOX2‐catalyzed ROS production, which promotes the formation of pMHC complexes. In addition, T‐MP increases ROS and activates the lysosomal Ca^2+^ channel Mcoln2, leading to Ca^2+^ release and transcription factor EB activation, thereby promoting CD80 and CD86 gene expression.^[^ ^169^ ^]^ Zhang et al. documented that tumor cell‐derived microparticles could effectively transfer DNA fragments to DCs, resulting in type I IFN production through the cGAS/STING‐mediated DNA‐sensing pathway.^[^ ^170^ ^]^ Type I IFN promotes DC maturation and the presentation of tumor antigens to CTLs. Ping et al. fabricated a nanovacine by coating neoantigen‐loaded PLGA nanoparticles with a cancer cell membrane. The nanovacine enabled selective delivery of neoantigens to resident DCs and promoted the secretion of chemokine C‐C motif ligand 2 (CCL2), CCL3, and C‐X‐C motif ligand 10 (CXCL10) from macrophages, further potentiating the transfer of DCs to dLNs (**Figure** **8**a–c), which led to initiation of antitumor immunity in a personalized manner.^[^ ^171^ ^]^ Li et al. exploited a functionalized DNA tetrahedron and further camouflaged it with a cancer cell membrane to form a nanoregulator named Td@Gox‐TsG@C, which caused a strong endoplasmic reticulum stress response, inducing immunogenic cell death (ICD) of tumor cells and generation of tumor immunogens. Tumor immunogens further promote DC maturation, T‐cell proliferation, and infiltration.^[^ ^172^ ^]^ In addition, exosomes, naturally derived extracellular vesicles, secreted by tumor cells or immune cells, have been applied to modify biomaterials. Exosomes can transfer membrane proteins to the target cell membrane in their natural form. Kim et al. prepared engineered exosomes (mVSVG‐Exo) to induce xenogenization of tumor cells. It is easier for xenogenizing tumors to be recognized as nonself or foreign by the host immune system. Thus, these exosome‐based biomaterials either increase their antigenicity or generate danger signals, which activate DCs to induce cross‐presentation of antigens to CD8^+^ T cells against cancer.^[^ ^173^ ^]^ Viruses can be recognized as non‐self, thereby leading to the initiation of an immune response. Oncolytic viruses can be designed to selectively replicate in cancer cells while leaving healthy cells unharmed. Viral replication results in tumor cell lysis and the release of tumor antigens in the TME. These tumor antigens can then be taken up by DCs and utilized to kill tumor cells. Fusciello et al. designed an artificially cloaked viral nanovaccine (ExtraCRAd) by wrapping the oncolytic virus with tumor cell membranes as antigenic sources.^[^ ^174^ ^]^ ExtraCRAd provided tumor antigens and immunostimulatory signals to DCs, resulting in efficient antitumor efficacy in preventive and therapeutic vaccination. Thus, cell‐derived biomaterials with distinctive biological properties have tremendous potential to promote the efficiency of DC‐mediated immunotherapy.

#### 5.4.2 Bacteria‐Derived Biomaterials

In addition to cell membranes directly enveloping nanoparticles, bacterial membranes can be used for encapsulating antigens because of their strong immunogenicity associated with pathogen‐associated molecular patterns (PAMPs). Ni et al. fabricated bacterial pathogen backbones to serve as a novel biomimetic vaccine (demi‐bacteria) with good biological safety profiles. The synergistic effects of intrinsic PAMPs, encapsulated CpG, and bacterial morphology elicited strong CTL responses. The researchers also demonstrated the prominent prophylactic effects of the demi‐bacteria against malignant tumors.^[^ ^44^ ^]^ Encapsulin (Encaps) is isolated from the thermophile Thermotoga maritima, and it is highly thermostable.^[^ ^178^ ^]^ Choi et al. established an encapsulant using genetically modified Encaps to incorporate the SIINFEKL peptide, which activated DC‐mediated antigen‐specific CTLs and suppressed melanoma tumors in vivo. Patel et al. prepared a multifunctional bacterial membrane‐coated nanoparticle (BNP), that can capture cancer antigens after radiation therapy, enhance antigen uptake by DCs, facilitate DC cross‐presentation, and stimulate an efficient CTL response.^[^ ^179^ ^]^ Zhang et al. constructed nanostructures assembled from *Escherichia coli* and tumor cells to efficiently deliver TAAs and induce DC maturation through the stimulator of the STING pathway.^[^ ^175^ ^]^ They prepared nanoDCs, which were synthesized by coating activated DC membranes on folic acid and ferrous ion self‐assembled nanostructures (Figure 8d). NanoDCs as nanovaccines effectively inhibited tumor growth and metastasis formation without obvious side effects. VNP20009 is a type of attenuated *Salmonella*, which can served as a hypoxic drug delivery system. Chen et al. engineered the *Salmonella* by decorating their surface with newly synthesized heptamethine cyanine dyes NHS‐N782 and JQ‐1 derivatives to obtain the delivery system (N‐V‐J), which can promote tumor targeted photothermal therapy and DC‐mediated magnified immunotherapy.

### 5.5 Stimulated Responsive Biomaterials

#### 5.5.1 pH‐Responsive Biomaterials

pH‐responsive biomaterials are attractive because their physicochemical and biological properties change with different pH values, which generally occurs when they are endocytosed by cells. pH‐responsive biomaterials can be designed by employing polymer building blocks to change their charge and/or hydrophilicity in response to environmental pH. Alteration of the charge and hydrophilicity can further cause structure variations of the biomaterials.^[^ ^180^ ^]^ Recently, pH‐responsive antigen‐loaded polymer nanoparticles have been widely designed for activating DCs.^[^ ^181^ ^]^ These pH‐responsive nanoparticles are stable at neutral pH but labile under acidic conditions; thus, they rapidly degrade and release antigens within the acidic endolysosomal compartments of DCs, resulting in controlled intracellular antigen release. Liu et al. confirmed that ammonium bicarbonate (NH_4_HCO_3_)‐containing PLGA nanoparticles can be used for pH‐responsive antigen delivery. The nanovaccine could efficiently be taken up by DCs and disrupted to release antigens via the reaction between H^+^ and NH_4_HCO_3_, resulting in antigen escape from the lysosome into the cytoplasm.^[^ ^182^ ^]^ Hu et al. developed pH‐responsive nanoparticles using tertiary amines of 2‐(diethylamino) ethyl methacrylate (DEAEMA) repeat units as cores and 2‐aminoethyl methacrylate as shells.^[^ ^183^ ^]^ The DEAEMA in the nanoparticle cores was largely uncharged at extracellular or cytosolic pH, at which point the particles were shrunken. However, the core tertiary amines ionized and the nanoparticles swelled at endolysosomal pH. The pH‐responsive nanoparticles can load and release OVA antigens in response to endolysosomal pH. Immune experiments demonstrated that DCs pulsed with OVA antigens carrying pH‐responsive nanoparticles evoked four‐fold more IFN‐*γ* secretion from CD8^+^ CTLs than DCs pulsed with pH‐nonresponsive nanoparticles. Some researchers have described that covalent tethering of antigens to pH‐responsive nanoparticles enhances intracellular antigen accumulation.^[^ ^184^ ^]^ Intracellular antigens can further promote the cross‐presentation of DCs and induce significantly high CTL responses. Wang et al. developed amphiphilic pH‐sensitive galactosyl dextran‐retinal (GDR) nanogels to load OVA antigens. The pH‐sensitive nanogels swelled to release OVA under acidic conditions, then eliciting ROS generation and enhancing the proteasome activities and MHC I antigen presentation of DCs. These nanogels boosted antigen uptake and cytosolic release and promoted DC maturation by activating retinoic acid receptor signaling.^[^ ^185^ ^]^

In addition to polymers, pH‐responsive inorganic materials can also be used in DC‐based vaccines. To avoid the degradation of internalized antigens in lysosomes, Wang et al. developed antigen‐doped CaCO_3_ nanomissiles, which can be enriched in acidic lysosomes of DCs (Figure 8e). Accompanied by the decomposition of the pH‐responsive CaCO_3_ nanomissiles, the generation of drastic CO_2_ caused rupture of the lysosomal membranes. Subsequently, the OVA antigen was released into the cytosol and further upregulated the expression of the OVA_257‐264_‐MHC I complex.^[^ ^176^ ^]^ The design of pH‐responsive biomaterials capable of altering their properties is of particular importance for DCs.

#### 5.5.2 Photochemically Responsive Biomaterials

Photochemical internalization (PCI) is a novel drug delivery strategy that enhances the delivery of immune molecules into the cytoplasm based on light and photosensitizers. The photosensitizer is selectively localized in endosomal or lysosomal membranes. PCI then triggers membrane rupture, which facilitates antigenic release and delivery.^[^ ^186^ ^]^ Zhang et al. prepared a biomaterial (PheoA‐PEI) by pheophorbide A grafted with polyethylenimine, which exhibited near‐infrared imaging and endosome escape properties. The complexed PheoA‐PEI‐OVA nanoparticles were responsive to light and could enhance cytosolic OVA antigen release to DCs, which promoted OVA‐specific CD8^+^ T‐cell immune responses.^[^ ^187^ ^]^ Hjalmsdottir et al. reported a PCI‐triggered cytosolic antigen delivery system that consisted of a photosensitizer tetraphenyl chlorine disulfonate (TPCS2a) and OVA antigen. The PCI‐triggered system promotes the antigen presentation process transition of DCs from MHC‐II to MHC‐I, facilitating IFN‐*γ* secretion by CD8^+^ CTLs.^[^ ^188^ ^]^ Therefore, photosensitive biomaterials also have the potential to activate DC vaccination.

### 5.6 “Carrier‐Free” Biomaterials

Synthetic peptides are the most commonly used forms of neoepitopes and have exhibited promising efficacy in antigen processing for DCs.^[^ ^189^ ^]^ However, antigenic peptides typically have a molecular weight of <5 kDa. Small peptides are easily cleared from systemic circulation and thus elicit minimum immune responses.^[^ ^190^ ^]^ To overcome the easy elimination of small antigenic molecules and minimize the potential risks of using additional carrier materials, Wei et al. developed a novel and versatile approach that designed a redox‐responsive polycondensate neoepitope (PNE) to activate DCs (Figure 8f).^[^ ^177^ ^]^ In this work, peptide neoantigens and adjuvants were combined with a traceless responsive linker monomer by a reversible polycondensation reaction. The redox‐responsive PNEs efficiently targeted and accumulated in dLNs. They significantly promoted antigenic capture and cross‐presentation by DCs. Each internalized carrier‐free nanoparticle contains thousands of specifically targeted antigen epitopes that are available for processing and presentation by DCs. Hao et al. documented that peptide‐crosslinked micelles significantly enhanced the uptake of antigenic peptides by human DCs. This result was attributed to the fact that the antigenic peptides were crosslinked and encapsulated and could, therefore, avoid degradation by serum components.^[^ ^191^ ^]^ Nanostructured immune‐polyelectrolyte (iPEM) capsules have been assembled through alternate deposition of antigenic peptides and TLR agonists.^[^ ^192^ ^]^ The iPEM capsules assembled entirely from polyionic immune signals, which eliminated the use of support materials, synthetic polymers, and other carrier components. Immunization with the iPEM capsules enhanced the expansion of antigen‐specific CD8^+^ T cells by promoting DC functions. Tsoras and Champion constructed peptide nanoclusters (PNCs) using cross‐linked antigenic peptides. These PNCs were readily internalized by DCs and could also passively diffuse into regional dLNs.^[^ ^51^ ^]^ Schetters et al. developed a novel conjugation platform of synthetic antigenic peptides (Antigen MAtriX). Tumor‐associated antigens and neoantigens were incorporated into the Antigenic MAtriX for DC targeting. They were shown to induce tumor‐specific effector CD8^+^ T‐cell responses.^[^ ^193^ ^]^

In addition to antigenic peptides, some other molecules can be used as “carrier‐free” ingredients. The antigenic protein molecule OVA antigen was encapsulated by coordination polymers of manganese (Mn^2+^) and meso‐2,6‐diaminopimelic acid (DAP), and a nucleotide oligomerization binding domain 1 (Nod1) agonist was used as the organic ligand.^[^ ^194^ ^]^ Wang et al. self‐assembled OVA antigens by exposing and crosslinking free thiols that had been embedded in hydrophobic regions. The nanovaccine comprised 97% OVA and 3% CpG agonist.^[^ ^195^ ^]^ It promoted DC‐based CTL responses that suppressed the growth of B16‐OVA melanoma. Zhu et al. described self‐assembled intertwining DNA‐RNA nanocapsules (iDR‐NCs) that incorporated stat3 short hairpin RNA (shRNA) and CpG via nucleic acid‐based nanotechnology. The iDR‐NC assemblies of both DNA and RNA therapeutics synergistically activated DCs, while iDR‐NC/neoantigen complexes elicited potent and durable tumor‐specific antitumor immunity.^[^ ^196^ ^]^ Chen and coworkers used cooperative *π*–*π* stacking and electrostatic interactions to produce a “carrier‐free” nanoassembly of a doxorubicin (DOX) prodrug and PD‐L1 siRNA (named PEG@D:siRNA). This PEG@D:siRNA simultaneously delivered the DOX prodrug and siRNA into tumor cells, which induced ICD of tumor cells and suppressed the upregulation of the PD‐L1 gene. Thus, PEG@D:siRNA further promotes the maturation of DCs and the activation and infiltration of CTLs to improve cancer chemoimmunotherapy.^[^ ^137^ ^]^ Mn^2+^ can activate cGAS‐STING signaling in DCs to elicit robust adaptive antitumor immunity.^[^ ^197^ ^]^ Zhang et al. developed a generally minimalist construction method of “carrier‐free” personalized tumor nanovaccine (PNV), which contained the supernatant of tumor abrasive fluid (STAF) antigen, CpG, and Mn^2+^.^[^ ^198^ ^]^ The PNV not only showed good tumor preventive effects but also successfully inhibited tumor development and metastasis. These studies provide preparation approaches for “carrier‐free” biomaterials to activate DCs, which increase the percentage of delivered antigens compared to other antigenic delivery systems with nonantigenic materials.

### 5.7 Microneedles

**Figure 9:**
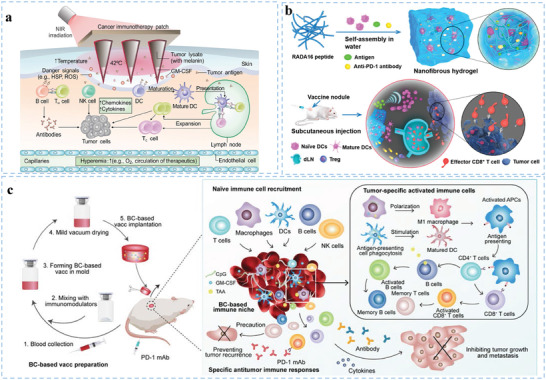
Microneedles and 3D scaffolds promoting DCs in tumor immunotherapy. a) Schematic illustration of microneedle‐based transdermal vaccination for activating DCs. Reproduced with permission.^[^ ^208^ ^]^ Copyright 2017, American Association for the Advancement of Science. b) Formation and mechanism of the DC‐based vaccine nodule engineered in the peptide nanofibrous hydrogel. Reproduced with permission.^[^ ^45^ ^]^ Copyright 2018, American Chemical Society. c) Schematic of the implantable blood clot vaccine. Reproduced with permission.^[^ ^214^ ^]^ Copyright 2020, American Association for the Advancement of Science.

There are a considerable number of DCs in the epidermis and dermis.^[^ ^199^ ^]^ Dermal DCs can capture peripheral antigens and quickly reach the dLNs to active T cells within 18 h after stimulation.^[^ ^200^ ^]^ Therefore, the skin is an attractive organ used as an immunization site. Microneedles are a newly developed effective minimally invasive biomaterial to facilitate antigen delivery through the skin.^[^ ^201^ ^]^ Microneedle skin patches can be designed to penetrate the outer layers of the stratum corneum and painlessly deposit vaccines in the epidermis and/or upper dermis.^[^ ^202^ ^]^ Microneedle skin patches include an array of solid pyramidal or cylindrical projections.^[^ ^203^ ^]^ Compared to traditional intramuscular injection, administration through microneedles has the advantage of targeting the abundant repertoire of immune cells in the skin by the vaccine. Therefore, they have been proposed for use in transdermal immunotherapy because they can directly transport antigens and immunomodulatory agents into the DC‐enriched niche of the dermis layer. In addition, it provides a way to encapsulate bioactive molecules in a stable, lyophilized state.^[^ ^204^ ^]^ The matrix of microneedles can be designed to be dissolved and consist of microparticles or nanoparticles carrying antigens or adjuvants. Therefore, antigen‐loaded particles can be rapidly deposited into the skin and the antigen release kinetics from the implanted particles can be controlled.^[^ ^205^ ^]^ In addition to the fact that microneedles are painless, easy to use, and exhibit a good targeting ability for skin DCs, they also have the advantage of being utilized as a safe immune stimulation platform that is capable of avoiding direct contact between the general circulation and adjuvants.^[^ ^206^ ^]^ It was reported that dissolving microneedle arrays that are laden with nanoencapsulated antigens increase vaccine immunogenicity by directly activating DCs within the skin.^[^ ^207^ ^]^ Ye et al. prepared a light‐activated transdermal microneedle patch, which can increase the localization of DCs in the skin after microneedle vaccination.^[^ ^208^ ^]^ Meanwhile, the microneedles not only promoted the activation of DCs in the dLNs, but also increased tumor‐infiltrating CTLs; thus, this immunotherapy strategy significantly prevented the growth of primary and distant tumors (**Figure** **9**a). Tu et al. developed an antigen delivery system by coating microneedle arrays with a pH‐sensitive pyridine layer and subsequently adsorbed lipid bilayer‐coated, OVA‐loaded mesoporous silica nanoparticles, named LB‐MSN‐OVA. This strategy enhanced OVA uptake by DCs.^[^ ^209^ ^]^ Maaden et al. explored OVA‐coated pH‐sensitive microneedle arrays as an effective vaccination platform,^[^ ^210^ ^]^ which induced robust CD8^+^ T‐cell responses. Lee et al. studied OVA antigen administration to the skin of mice using a dissolving microneedle.^[^ ^211^ ^]^ The researchers documented that the administration strategy evoked OVA‐specific CTL responses, which inhibited grafting of E.G7‐OVA tumor cells in the immunized mice. Zeng et al. developed immune polyelectrolyte multilayer (iPEM)‐coated microneedle arrays based on the self‐assembly of human melanoma antigens and CpG. It was shown that the cargo released from the microneedle can be internalized by primary DCs, generating tumor‐specific immune responses.^[^ ^212^ ^]^ Naito et al. documented that antigen‐loaded dissolving microneedle arrays effectively delivered substantial amounts of OVA into the skin within three minutes and induced robust antigen‐specific CTL responses by activating immunocompetent DCs.^[^ ^213^ ^]^ Microneedle development inevitably provides a promising direction for the future clinical transformation of DC vaccines.

### 5.8 3D Scaffolds

3D scaffolds can create a highly defined microenvironment, deliver antigens into mDCs, and minimize tumor‐induced tolerance, which is extremely important for therapeutic immune responses.^[^ ^215^ ^]^ 3D scaffolds have been proven to be a favorable tool, as they present DCs with activating cues in a sustained manner at a localized site.^[^ ^216^ ^]^ Ali et al. implanted resorbable poly‐lactide‐co‐glycolide (PLG) polymer sponges into the skin and showed that these porous vaccine scaffolds promoted the attraction, activation, and antigen loading of DCs by controlling the release kinetics of GM‐CSF at the implant site of the scaffold.^[^ ^217^ ^]^ The mDCs then leave the scaffold and migrate to the dLNs to activate naïve T cells. Additionally, researchers developed a 3D, porous polymer matrix that was loaded with tumor lysates.^[^ ^218^ ^]^ 3D scaffolds with mitoxantrone‐treated tumor cells elevate DC migration and enhance their maturity and proliferation in the 3D microenvironment.^[^ ^219^ ^]^ These matrices presented distinct combinations of GM‐CSF and TLR agonists that achieved 70% to 90% prophylactic tumor protection in B16‐F10 melanoma models. Kim et al. demonstrated that mesoporous silica rods (MSRs) with a high‐aspect ratio accumulate spontaneously after injection into subcutaneous tissue. The mesoporous silica rods then form a macroporous structure that provides a 3D cellular microenvironment for host DCs.^[^ ^220^ ^]^ Bencherif et al. subcutaneously implanted GM‐CSF and CpG oligodeoxynucleotides (ODNs) in spongy macroporous cryogels into mice to induce local infiltration of DCs.^[^ ^221^ ^]^ This strategy induced an effective, durable, and specific antitumor T‐cell response in melanoma models. Yang et al. developed vaccine nodules by encapsulating DCs and tumor antigens into a self‐assembled nanofibrous peptide hydrogel.^[^ ^45^ ^]^ The nanofibrous hydrogel, as a 3D matrix, enhanced the survival time of encapsulated DCs (Figure 9b). The vaccine nodules can also recruit abundant host DCs and promote the drainage of activated DCs to dLNs. In addition, Singh et al. exploited injectable, in situ‐forming, and biodegradable hydrogels to deliver DC‐attracting chemokines.^[^ ^222^ ^]^ The chemokine‐carrying gels recruited 4–6‐fold more DCs than the control gels. In patients with B‐cell lineage malignant non‐Hodgkin's lymphomas, in situ cross‐linkable chemokine‐carrying hydrogels were found to recruit iDCs in muscles.^[^ ^223^ ^]^ The immune‐priming hydrogels attracted DCs, modulated cytokine signaling, and evoked a Th1‐based immune response to plasmid‐encoded antigens. Locally delivered engineered 3D polymer scaffolds acting as synthetic immune niches can boost anticancer immunity, modulate local immunity, enable effective treatment at lower doses, and prevent systemic toxicity.^[^ ^224^ ^]^ RBCs have been used to construct a scaffold for modulating the innate immune system. Recently, Qin and coworkers developed an implantable natural blood clot scaffold from autologous blood for tumor‐associated antigen and adjuvant (CpG) delivery and found that the blood clot attracts and recruits DCs due to its intrinsic immune‐stimulating effects (Figure 9c). These blood clot vaccines (BCVs) lead to the formation of an “immune niche” and then induce a robust anticancer immune response.^[^ ^214^ ^]^

Overall, engineered 3D scaffolds can improve the viability of DCs and maintain their biological function, which promotes antigen uptake, activation, maturation, and lymph node migration of DCs, thus stimulating a strong tumor‐specific immune response.

## 6 Engineered Biomaterials Reprograming Immunosuppressive Cells in TME

**Figure 10:**
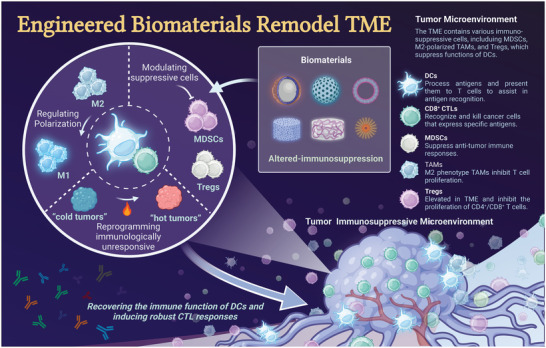
Reprogramming the immunosuppressive cells in TME. The TME contains various immunosuppressive cells, including MDSCs, M2‐polarized TAMs, and Tregs, which suppress the functions of DCs. Engineered biomaterials can recover the immune function of DCs by modulating suppressive cells (e.g., MDSCs and Tregs), regulate polarization of TAMs, and convert a cold TME to a hot TME, thus facilitating robust CTL responses.

The TME contains abundant immunosuppressive cells, including MDSCs, M2‐polarized TAMs, and Tregs, which establish a comprehensive interaction and inhibit the functions of DCs through nutrition depletion, phenotype alternation, apoptosis and anergy,^[^ ^225^ ^]^ thus hindering DC‐mediated normal immune responses. Reprogramming the immunosuppressive cells in the TME offers promising strategies for recovering the immune function of DCs (**Figure** **10**).

### 6.1 Biomaterials Modulating MDSCs and Tregs

**Figure 11:**
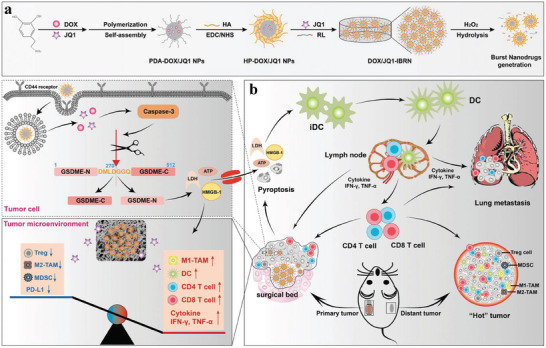
Biomaterials reprograming immunosuppressive cells in TME. a) Preparation procedure and degradation of an implantable bioresponsive immunotherapeutic nanoarray, and b) schematic illustration showing the immunological effects of the nanoarray. Reproduced with permission.^[^ ^229^ ^]^ Copyright 2020, Wiley‐VCH.

MDSCs express elevated levels of PD‐L1, which promotes tumor proliferation and metastasis by inhibiting T‐cell immunity.^[^ ^226^ ^]^ A variety of biomaterials have been engineered to address the immunosuppressive effects of MDSCs and Tregs in the TME. Kempe et al. designed mannosylated hollow glycopolymer microcapsules that can upregulate CD80 on DCs and downregulate PD‐L1 on MDSCs. These effects simultaneously promoted DC functions and inhibited MDSCs.^[^ ^227^ ^]^ Chen et al. designed P/PEALsiCD155 polymeric nanoparticles for PD‐L1 and CD155 asynchronous blockades, which synergistically enhanced the DC maturation percentage and reduced the percentage of Tregs.^[^ ^228^ ^]^ As shown in **Figure** **11**a,b, Zhao et al. designed an implantable bioresponsive nanoarray (DOX/JQ1‐IBRN) for modulating the immunosuppressive TME. By facilitating immunogenicity, blocking the immunosuppressive PD‐L1 pathways, and modulating immunosuppressive cells (Tregs, MDSCs), DOX/JQ1‐IBRN can transfer “cold tumors” to “hot tumors”, thus facilitating DC effects and avoiding immune evasion.^[^ ^229^ ^]^

### 6.2 Biomaterials Regulating the Polarization of Macrophages

**Figure 12:**
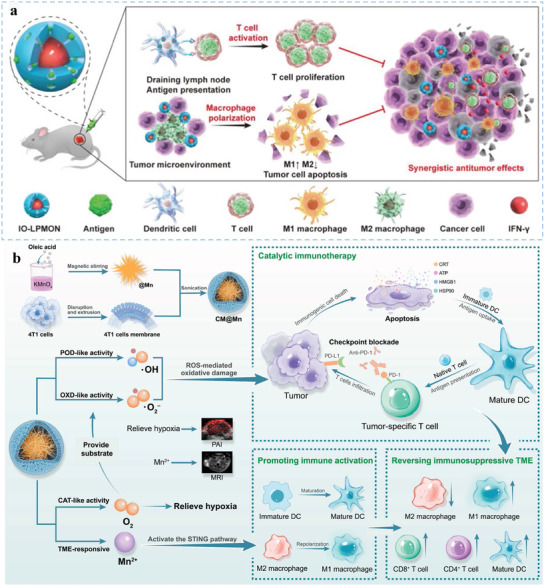
Biomaterials regulating the polarization of macrophages in TME. a) Biomaterials regulate tumor‐associated macrophage polarization. Schematic illustration of the simultaneous DC activation and macrophage polarization for potent antitumor immunotherapy using IO‐LPMONs. Reproduced with permission.^[^ ^230^ ^]^ Copyright 2019, Wiley‐VCH. b) Schematic illustration of the process of preparation of CM@Mn nanozyme and the therapeutic strategy of TME‐activable manganese‐boosted catalytic DC‐mediated immunotherapy. Reproduced with permission.^[^ ^235^ ^]^ Copyright 2022, American Chemical Society.

Macrophages can be polarized into M1 or M2 phenotypes that have different roles. TAMs of the M2 phenotype promote tumor proliferation, which is often associated with poor prognosis in many cancer types.^[^ ^231^ ^]^ Recently, it was reported that TAMs can be polarized from an immunosuppressive M2 phenotype toward an antitumor M1 phenotype by biomaterials.^[^ ^232^ ^]^ Biomaterial‐mediated polarization of macrophages in the TME enhances cancer immunotherapeutic efficacy.^[^ ^233^ ^]^ Chen et al. designed and developed iron oxide‐embedded large‐pore mesoporous organosilica nanospheres (IO‐LPMONs) that simultaneously promoted the cross‐presentation of DCs and M1‐polarization of TAMs for potent antitumor immunotherapy[230] (**Figure** **12**a). Chloroquine (CQ) has been used as an efficient immunomodulator for TAM polarization. Liu et al. described that quantum dot (QD)‐pulsed DCs in combination with CQ ameliorated the immunosuppressive TME by polarizing TAMs into M1 macrophages, thereby evoking a strong antitumor immune response.^[^ ^234^ ^]^ Zhao et al. constructed a nanovaccine (CM@Mn) with intrinsic peroxidase and oxidase‐like activity properties in an acidic TME (Figure 12b).^[^ ^235^ ^]^ CM@Mn can not only evoke ICD of tumors in the TME by generating toxic hydroxyl (•OH) and superoxide radicals (•O^2‐^) but also release Mn^2+^, which directly promotes DC maturation and macrophage M1 repolarization to reverse the immunosuppressive TME into an immune‐activating environment. Therefore, TAMs can be polarized using biomaterials in the TME to ensure the effect of DC‐based immunotherapy.

## 7 Engineered Biomaterials in Combination with Other Antitumor Therapies

**Figure 13:**
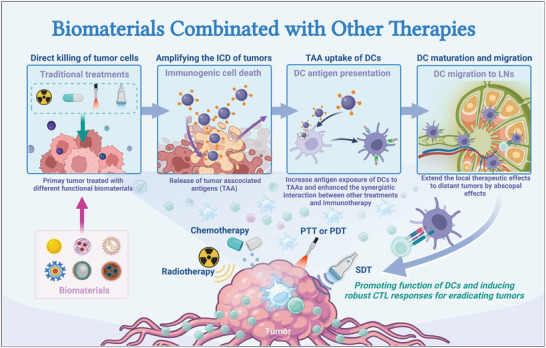
Biomaterials in combination with other therapies. The TME limits immunotherapeutic effects. Many traditional treatment modalities, such as radiotherapy, chemotherapy, PTT, PDT, and SDT, have been applied in combination with biomaterial‐mediated DC immunotherapies. These ablative treatment methods can be amplified, and then produce TAAs for DCs to induce tumor‐specific immune responses. Immunotherapy combination strategies extend the local therapeutic effects to distant tumors by abscopal effects. Thus, the combination of traditional treatment modalities with DC‐based immunotherapies can exert a powerful treatment effect.

The survival rate and immunotherapy response of cancer patients are closely correlated with the complexity of the TME, immuno‐score, and immune context of tumors.^[^ ^236^ ^]^ Relatedly, only a minority of cancer patients benefit from current immunotherapies. The TME limits immunotherapeutic effects. Many traditional treatment modalities, such as radiotherapy, chemotherapy, PTT, PDT, and SDT, have been applied in cancer therapies.^[^ ^237^ ^]^ However, these modalities alone exhibit a limited therapeutic effect. These ablative treatment methods are able to induce tumor‐specific immune responses by producing TAAs. The combination of traditional treatment modalities with DC‐based immunotherapies can exert a powerful treatment effect. Thus, the development of biomaterials that benefit combination therapies holds great promise for tumor therapy because they not only improve efficacy but also overcome side effects (**Figure** **13**). The exemplified biomaterials combinated with other antitumor therapies for promoting DC‐mediated immunotherapy are listed in **Table** **3**.

**Table 3:** Representative biomaterials combinated with other antitumor therapies for promoting DC‐mediated immunotherapy (Abbreviations: AC‐NPs, antigen‐capturing nanoparticles; TDPAs, tumor‐derived protein antigens; Cat, catalase; CM@MON@KP1339, cell membrane vesicles of tumor cells coated with MON@KP1339; APNA, photothermally activatable polymeric pro‐nanoagonist; PVP‐MPDA@R837, imiquimod loaded into mesoporous polydopamine and modified by polyvinyl pyrrolidone; AM, AuNC@MnO_2_; C@HPOC, hybrid protein oxygen nanocarrier with chlorine e6 encapsulated; HMME, hematoporphyrin monomethyl ether; PIMS NPs, phenolic nanoadjuvant; N/A, not applicable)

No. ^[Ref.]^	Other antitumor therapies	Biomaterials	Tumor model	Mechanisms of DC modulations	Synergistic antitumor effects
1[60b]	Radiotherapy	AC‐NPs	B16F10	Increased exposure of DCs to tumor‐specific antigens that are released after radiotherapy‐induced tumor‐cell death	After treated with radiotherapy, AC‐NPs captured the TDPAs released during radiotherapy and transporting them to DCs, and thereby promoted antitumor immunity.
2[41b]	Radiotherapy	PLGA‐R837@Cat	CT26	Enhanced the ICD for tumor cells and generated tumor debris as antigen; Promoted DC maturation	After treated with radiotherapy, PLGA‐R837@Cat induced strong DC‐mediated antitumor immune responses, inhibited tumor metastases and protected mice from tumor re‐challenging.
3[238]	Chemotherapeutics	DOX/R837/ALG or OXA/R837/ALG + *α*PD‐L1	CT26	Enhanced the ICD of tumor and promoted DC maturation	Localized chemotherapy with ICD drugs, together with DC adjuvants and ICBs, synergistically triggers a robust systemic antitumor therapeutic outcome with reduced systemic toxicity.
4[239]	Chemotherapeutics	CM@ MON@KP1339	4T1	Evoked oxidative and ER stress in parallel for the induction and amplification of ICD and promoted DC activation	CM@MON@KP1339 amplified ICD and boosted robust antitumor immunity for regression of both primary and distant tumors.
5[240]	Photothermal therapy	PVP‐MPDA@R837	B16F10	Promoted LNs‐targeted DC activation	Combining photothermal conversion property of PVP‐MPDA@R837 with lymphatic‐focused immune activation led to the growth inhibition of tumor.
6[241]	Photothermal therapy	APNA	4T1	Generated superior T‐cell stimulation capacity of pre‐activated APNA‐treated DCs	APNA under NIR‐II light potentiates systemic antitumor immunity, leading to promoted DC‐mediated CTLs and helper T‐cell infiltration in distal tumor, lung and liver to inhibit cancer metastasis.
7[242]	Photodynamic therapy	AM	4T1	Promoted DC maturation, and subsequently induced prominent activation of specific effector cells	The oxygen‐boosted PDT effect of AM not only destroys primary tumor effectively but also elicits ICD, thereby robustly evoking systematic antitumor immune responses.
8[243]	Photodynamic therapy	C@HPOC	4T1	Oxygen‐boosted PDT of C@HPOC induced ICD, with the release of DAMPs to activate DCs	C@HPOC‐mediated immunogenic PDT could destroy primary tumors and effectively suppress distant tumors and lung metastasis by evoking systemic antitumor immunity.
9[244]	Sonodynamic therapy	HMME/R837@Lip	4T1/CT26	Promoted DCs maturation and cytokine secretion	HMME/R837@Lip with SDT not only suppresses the primary tumors but also mitigate the progression of tumor metastasis, and protects against tumor re‐challenge.
10[245]	Sonodynamic therapy	PIMS NPs	4T1	Enhanced DCs maturation via combinational action of SDT‐mediated ICD effect and Mn2+ promoted the activation of the cGAS‐STING pathway	PIMS NPs with SDT enhanced inhibition for both primary and distant tumor growths, and greatly restrained lung metastasis.

### 7.1 Combination of Radiotherapy with DC‐Mediated Immunotherapy

**Figure 14:**
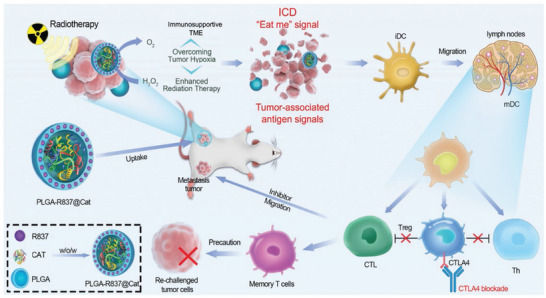
Biomaterials for combined radiotherapy and DC‐mediated immunotherapy. Biomaterials for combined radiotherapy to promote DC‐mediated immunotherapy. Schematic illustration of the mechanism of DC‐mediated antitumor immune responses induced by PLGA‐R837@Cat‐based radiotherapy to inhibit cancer metastases and recurrence. Reproduced with permission.^[^ ^41b^ ^]^ Copyright 2019, Wiley‐VCH.

The combination of radiotherapy with immunotherapy induces an abscopal effect that produces systemic regression of metastatic lesions.^[^ ^246^ ^]^ Min et al. developed several antigen‐capturing nanoparticles (AC‐NPs) that significantly improved the abscopal effect via combined radiotherapy.^[^ ^60b^ ^]^ In this work, injected AC‐NPs with functionalized surfaces captured antigens released from the tumor receiving radiotherapy, and trafficked to nearby tumor‐draining lymph nodes (TDLNs) through DC‐mediated transport. Subsequently, the AC‐NPs promoted CD8^+^ CTL expansion and increased the CD4^+^ T‐cell/Treg and CD8^+^ T‐cell/Treg ratios. Chen et al. reported a PLGA‐based core–shell nanostructure encapsulated with catalase (CAT) and R837 (**Figure** **14**), which can greatly strengthen radiotherapy efficacy by modulating the TME and promoting DC presentation.^[^ ^41b^ ^]^ Ni et al. reported porous Hf‐based nanoscale metal‐organic frameworks (nMOFs) as highly effective radio‐enhancers.^[^ ^247^ ^]^ nMOF‐mediated low‐dose radiotherapy effectively destroyed the irradiated tumors, caused ICD, and released tumor antigens for DC cross‐presentation. Therefore, it extends the local therapeutic effects of radiotherapy to distant tumors via abscopal effects. Radiation‐induced pro‐inflammatory protein production and increased exposure of DCs to tumor antigens enhance the synergistic interaction between radiotherapy and immunotherapy. The TME is typically immunosuppressive and the release of tumor antigens mediated by radiotherapy does not sufficiently activate sufficient DC immune responses. Wang et al. reported an emerging strategy for inducing the migration of antigens out of the immunosuppressive TME to then activate DCs.^[^ ^248^ ^]^ Specifically, they have used bacteria (*Salmonella*) to adsorb antigens produced by radiotherapy and then deliver the antigens from the tumor into the tumor marginal tissue to activate DCs. This simple strategy makes immune initiation relatively easier regardless of the suppressive factors in the tumor. In addition, radiotherapy is a type of oxygen‐consuming tumor treatment, the limited intratumoral oxygen contents would limit the production of cytotoxic ROS and restrict the therapeutic efficacy of radiotherapy. Dong et al. reported a nanoregulator (PFCE@fCaCO_3_‐PEG) that could improve the therapeutic outcome of radiotherapy. The PFCE@fCaCO_3_‐PEG can reverse tumor immunosuppression and potentiate radiotherapy through chemically modulating tumor hypoxic and acidic microenvironments.^[^ ^246b^ ^]^

### 7.2 Combination of Chemotherapy and DC‐Mediated Immunotherapy

**Figure 15:**
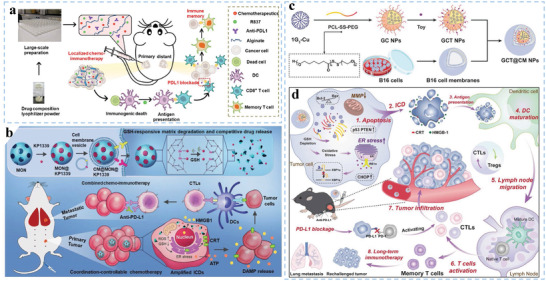
Biomaterials for DC‐related chemoimmunotherapy. a) Scheme illustrating local chemoimmunotherapy using various drug composites. Reproduced with permission.^[^ ^238^ ^]^ Copyright 2020, American Association for the Advancement of Science. b) Biomaterials for combined chemotherapy to promote DC‐mediated immunotherapy. Schematic of the synthesis of diselenide bond‐bridged MONs for coordination‐responsive drug release and amplified ICD for efficient and safe cancer chemoimmunotherapy. Reproduced with permission.^[^ ^239^ ^]^ Copyright 2021, Wiley‐VCH. c) Schematic illustration of the preparation of GCT@CM NPs. d) Cooperative tumor suppression through GCT@CM NP‐mediated chemotherapy for enhanced DC‐mediated immunotherapy. Reproduced with permission.^[^ ^250^ ^]^ Copyright 2022, Wiley‐VCH.

Chemotherapy that uses cytotoxic drugs to kill tumor cells is a conventional treatment method for cancer. However, many chemotherapeutic drugs that target fast‐dividing cells may result in immunosuppression of the body or toxic side effects.^[^ ^249^ ^]^ The combination of chemotherapeutics and immunotherapy using engineered biomaterials can amplify chemotherapy‐driven ICD, which is an efficient and safe antitumor method. Chemotherapy can induce ICD of tumor cells and upregulate the expression or release of DAMPs, such as adenosine triphosphate (ATP), calreticulin (CRT), and high‐mobility group box1 protein (HMGB1).^[^ ^251^ ^]^ ATP functions as chemoattractant signal for DC precursors, while CRT acts as an “eat me” signal to promoting phagocytosis of DCs and trigger antigen‐specific T‐cell responses.^[^ ^252^ ^]^ Meanwhile, liberated HMGB1 activates TLR‐4 to stimulate DCs maturation.^[^ ^253^ ^]^ Therefore, combination of chemotherapy and DC‐mediated immunotherapy may potentiate the therapeutic efficacy by killing tumor cells and augmentation of host immune system.^[^ ^254^ ^]^ Fan et al. first utilized mitoxantrone to induce ICD of tumor cells and release immunostimulatory ligands. Subsequently, they applied nano‐depot platform to load immunostimulatory ligands and conjugate them with immunogenically dying tumor cells. The conjugation efficiently promoted antigen presentation by DCs and elicited strong antitumor immune responses in melanoma and colon carcinoma.^[^ ^255^ ^]^ Some biomaterials can induce ICD of tumor cells as chemotherapeutic drugs, which can be combined with immunotherapy for tumor treatment. Yang et al. designed Cu^2+^‐doped dendritic mesoporous organosilica nanoparticles (Cu‐DMONs) for chemo‐immunotherapy.^[^ ^256^ ^]^ On the one hand, the doped Cu^2+^ induced ICD of tumor cells via Fenton‐like reactions. On the other hand, the Cu‐DMONs exhibited intrinsic immune‐adjuvant activities that stimulated DC maturation. Chao et al. designed a “cocktail” chemoimmunotherapeutic composite containing an ICD‐inducing chemotherapeutic drug (DOX or oxaliplatin), TLR agonists (R837), and a pharmaceutical excipient alginate for localized chemoimmunotherapy (**Figure** **15**a).^[^ ^238^ ^]^ The cocktail therapeutic composites induced slow release of chemotherapeutic drugs and immune adjuvants, effectively promoting DC maturation in LNs. Liang et al. described a novel strategy that combines chemotherapy and immunotherapy to modulate the TME by systemically and concurrently delivering chemotherapeutic agents (SN38) and a STING agonist (DMXAA) into tumors using triblock copolymer nanoparticles as a carrier. This strategy not only showed chemotherapeutic effects on tumor cells but also enhanced the antigenic cross‐presentation of DCs and converted the immunosuppressive TME into an immunogenic TME.^[^ ^257^ ^]^ Zhang et al. designed diselenide‐bridged mesoporous organosilica nanoparticles (MONs) for loading chemotherapeutic ruthenium compounds (KP1339). The constructed MSN@KP1339 significantly increased the frequency of DC maturation and secretion levels of TNF‐*α*, IFN‐*γ*, and IL‐6, which indicated that MON@KP1339 efficiently enhanced tumor immunogenicity by promoting DC maturation (Figure 15b). MSN@KP1339 also selectively evoked reactive oxygen species (ROS) production, glutathione depletion, and endoplasmic reticulum stress in cancer cells, thus amplifying the ICD induced by KP1339 and boosting robust immune responses.^[^ ^239^ ^]^ Guo et al. designed cell membrane‐camouflaged and 1G_3_‐Cu/Toy‐loaded polymeric nanoparticles for highly efficient chemotherapy‐potentiated DC‐mediated immunotherapy.^[^ ^250^ ^]^ The constructed biomaterials enable the responsive release of dual drugs in the TME and amplify the ICD of tumors through both mitochondrial and ER pathways, resulting in DC maturation and CTL recruitment (Figure 15c,d).

### 7.3 Combination of Photothermal Therapy and DC‐Mediated Immunotherapy

**Figure 16:**
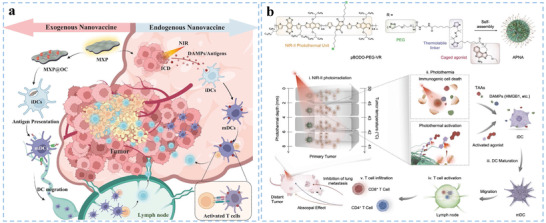
Biomaterials Used for Combined PTT. a) Schematic illustration of the MXP nanoplatform as photothermal nanoagents and immune vaccines synergistically activating the DC‐based antitumor immune cascade to achieve effective tumor destruction. Reproduced with permission.^[^ ^261^ ^]^ Copyright 2023, Wiley‐VCH. b) Biomaterials used for combined PTT. Scheme of preparation and antitumor activity of photo‐responsive APNA by NIR‐II photothermal immunotherapy. Reproduced with permission.^[^ ^241^ ^]^ Copyright 2021, Springer Nature.

Photothermal therapy (PTT) is an emerging therapy that utilizes near‐infrared (NIR) light to irradiate light‐absorbing biomaterials accumulated in the tumor to convert light energy into heat energy for the thermal ablation of cancer cells.^[^ ^258^ ^]^ However, it is difficult to completely eradicate tumors with PTT alone because of the limited penetration depth of NIR light and the short period of laser irradiation, which can lead to tumor relapse and metastasis. Combining PTT with DC‐based immunotherapy is expected to overcome the above challenges.^[^ ^259^ ^]^ AuNPs have been used in cancer PTT for ablating accessible tumors. Pan et al. designed ICG‐OVA for NIR fluorescence imaging, PTT, and DC immunotherapy.^[^ ^260^ ^]^ They intratumorally injected ICG‐OVA followed by 808 nm laser irradiation, which increased tumor temperature and simultaneously promoted the maturation of DCs, thus suppressing the growth of B16 tumors. Zhang et al. reported a combined strategy by using Ti_3_C_2_ MXene‐based nanoplatforms (MXP) to promote the tumor eradication efficiency of PTT and DC‐based immunotherapy. MXP nanoplatform as a photothermal nanoagent and immune vaccine synchronously activating the DC‐based antitumor cascade immune response (**Figure** **16**a).^[^ ^261^ ^]^ In addition, Zhang et al. developed a novel cell vesicle composition‐coated AuNP (AuNP@DCB16F10). AuNP@DCB16F10 was prepared by sequentially incubating the AuNPs with murine melanoma cells and DCs, which retained tumor antigens and DC‐derived components. AuNP@DCB16F10 not only eradicated the primary tumor but also provoked antitumor immune responses, thereby suppressing tumor metastasis and recurrence.^[^ ^262^ ^]^ With advantages based on mDC cell membranes, Sun et al. prepared intelligent DCs, which consist of biomaterials loaded with photothermal agents (IR‐797) and coated with mDC membranes.^[^ ^263^ ^]^ Intelligent DCs can reduce the expression of heat shock proteins (HSPs) in tumor cells, making them more sensitive to PTT, therefore inducing long‐term and large‐scale thermal therapy.

Biomaterials loaded with TLR agonists are also employed in photothermal immunotherapy. Chen et al. prepared polydopamine (pD)‐coated Al_2_O_3_ (pD‐Al_2_O_3_) for CpG‐enhanced photothermal immunotherapy.^[^ ^264^ ^]^ By combining CpG, pD‐Al_2_O_3_ induces the maturation of DCs and effectively inhibits the recurrence and metastasis of tumors. Wang et al. prepared polyvinyl pyrrolidone (PVP) modified with R837‐loaded mesoporous polydopamine nanoparticles (PVP‐MPDA@R837), which not only carried adjuvants but also acted as photothermal agents. The PVP‐MPDA@R837+NIR treatment group exhibited a high maturation level of DCs in dLNs and the strongest inhibitory effect on B16F10 tumor growth.^[^ ^240^ ^]^ Jia et al. prepared self‐assembled nanoparticles containing indocyanine green (ICG, a photothermal agent), R848 (TLR‐7/8 agonist) and CpG (TLR‐9 agonist) and subsequently encapsulated them in a thermosensitive hydrogel. The researchers demonstrated that the composite hydrogel combined with NIR promoted the secretion of TNF‐*α* and IL‐6 and increased the population of mDCs. After tumor resection, the composite hydrogel was injected at the surgical site followed by laser irradiation (808 nm, 5 min), and nearly four‐fifths of the mice were completely cured without recurrence or lung metastatic nodules.^[^ ^265^ ^]^ Photothermal tumor ablation combined checkpoint‐blockade molecules overcomes several critical issues in cancer immunotherapy.

To prevent the recurrence and metastasis of tumors, biomaterials and ICBs have been combined with PTT for DC‐based immunotherapy. Chen et al. prepared multifunctional nanoparticles encapsulating both NIR light heaters and R837, which combined with CTLA‐4 checkpoint blockades could eliminate primary tumors, attack and kill spreading metastatic tumor cells, and offer immune‐memory protection to prevent tumor relapse.^[^ ^266^ ^]^ Wang et al. reported that photothermal ablation of primary tumors with single‐walled carbon nanotubes (SWNTs), which are used as an immune adjuvant, promoted DC maturation and the production of antitumor cytokines.^[^ ^267^ ^]^ SWNT in combination with the anti‐CTLA‐4 antibody further prevented the development of tumor metastasis in mice.

NIR‐II light (1000–1300 nm) possesses even better biological transparency and limited phototoxicity, although fewer NIR‐II nanotransducers are available. Jiang et al. prepared a photothermal activatable polymer nanoagonist (APNA) using NIR‐II light for combined PTT immunotherapy.^[^ ^241^ ^]^ After NIR‐II light exposure, APNA induced tumor ablation and ICD while liberating R848 to activate DCs. NIR‐II irradiation exposure to APNA induced a higher proportion of mDCs in dLNs in vivo, thus enhancing tumor‐infiltrating T cells to inhibit tumors (Figure 16b).

Some multifunctional PTT biomaterials have also been designed and developed. Yang et al. fabricated erythrocyte membrane‐camouflaged “nanobullets” using a thermal‐sensitive nitric oxide (NO) donor S‐nitrosothiols (SNO)‐pendant copolymer (poly(acrylamide‐co‐acrylonitrile‐co‐vinylimidazole)‐SNO copolymer, PAAV‐SNO), which was used to deliver NIR‐II photothermal agent IR1061 and indoleamine 2,3‐dioxygenase 1 (IDO‐1) inhibitor 1‐methyl‐tryptophan (1‐MT). The “nanobullets” can comprehensively reprogram the suppressive TME and switch immune “cold” tumors to “hot” tumors by interfering with IDO‐1 activity via 1‐MT and normalizing the tumor vessels via the effects of NO generated in situ upon laser irradiation.^[^ ^268^ ^]^ In addition, the “nanobullets” plus NIR‐II laser irradiation induced ICD, invoked an “eat‐me” signal for DC uptake, activated the immune system, and promoted the antitumor effect of IR1061‐mediated PTT.

Overall, biomaterials encapsulating PTT agents play important roles in facilitating ICD, promoting antigen uptake by DCs, and enhancing antitumor efficacy. Although, some novel biomaterials can mediate local thermal ablation of tumors and induce ICD by microwave ablation,^[^ ^269^ ^]^ the combination of PTT with DC‐based immunotherapy has still shown great potential in fighting cancer.

### 7.4 Combination of Photodynamic Therapy and DC‐Mediated Immunotherapy

**Figure 17:**
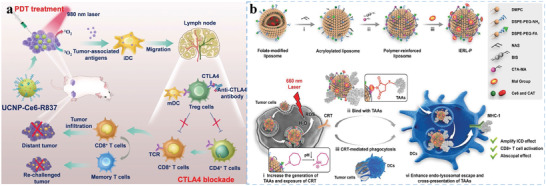
Biomaterials used for combined PDT. a) Scheme summarizing the combination mechanisms of NIR‐mediated photodynamic immunotherapy. Reproduced with permission.^[^ ^60a^ ^]^ Copyright 2017, American Chemical Society. b) IERL‐Ps enhance PDT‐induced ICD and systemically improve DC mediated antitumor responses. Reproduced with permission.^[^ ^275^ ^]^ Copyright 2022, Wiley‐VCH.

PDT can kill tumor cells utilizing photosensitizers (PSs) to generate ROS under light irradiation.^[^ ^270^ ^]^ Although PDT has advantages such as spatiotemporal selectivity and minimal invasiveness, conventional photosensitizers have shortcomings, including limited tumor tissue penetration, aggregation‐caused quenching, and phototoxicity; moreover, tumor hypoxia weakens the efficacy of oxygen‐dependent PDT.^[^ ^271^ ^]^ Biomaterials not only directly kill tumor cells with advantages in great spatiotemporal selectivity and minimal invasiveness, but also promote DC immune function for tumor inhibition via producing antigens from tumor cell residues. Xu et al. utilized NIR‐triggered PDT to enhance DC‐based antitumor therapeutic effects (**Figure** **17**a).^[^ ^60a^ ^]^ The researchers used lanthanide‐containing upconversion nanoparticles (UCNPs) as a platform for co‐loading of chlorin e6 (Ce6) and R837. The UCNP‐Ce6‐R837 biomaterials induced photodynamic destruction of the primary tumor after exposure to 980 nm NIR laser irradiation. After PDT, TAAs were generated and combined with UCNP‐Ce6‐R837, which effectively triggered DC maturation. Furthermore, combination of the immune‐stimulating UCNP‐based PDT strategy with CTLA‐4‐targeted therapy effectively inhibited distant tumor growth and prevented tumor recurrence through memory T cells. Liang et al. designed core–shell gold nanocage@manganese dioxide (AM) nanoparticles as TME‐ responsive oxygen producers and NIR‐triggered ROS generators for oxygen‐boosted immunogenic PDT against metastatic triple‐negative breast cancer.^[^ ^242^ ^]^ The oxygen‐boosted PDT effect of AM not only destroys primary tumor but also elicits ICD with DAMP release, which subsequently induces DC maturation. Chen et al. developed a hybrid protein oxygen nanocarrier with encapsulated Ce6 (C@HPOC) for oxygen self‐sufficient PDT.^[^ ^243^ ^]^ C@HPOC‐mediated immunogenic PDT could kill primary tumors and effectively suppress distant tumors and lung metastasis by activating DCs, CTLs, and NK cells in vivo. Ni et al. exploited an nMOF (W‐TBP) loaded with CpG, which is used to facilitate antigen presentation by enabling immunogenic PDT and inducing mDCs.^[^ ^272^ ^]^ Xu et al. constructed novel biodegradable mesoporous silica nanoparticles (bMSNs), which can deliver PSs for PDT, then recruited DCs to PDT‐treated tumor sites and elicited antigen‐specific CTLs.^[^ ^273^ ^]^ Kim et al. designed a silica nanocarrier decorated with a PEGylated azobenzene linker, which was loaded with CpG and glycol chitosan (CAGE). After treatment with CAGE‐mediated PDT, tumor cells can produce neoantigens and subsequently improve the antigen presentation activity of DCs.^[^ ^274^ ^]^ Zhao et al. reported an immune‐enhancing polymer‐reinforced liposome (IERL) that could promote TAA cross‐presentation in DCs and demonstrated its ability to amplify ICD‐associated antitumor immunity and improve the antitumor efficacy of PDT when loaded with Ce6 and catalase (Figure 17b).^[^ ^275^ ^]^ More exploration of the relationship between PDT and DC immunotherapy is still needed, and more advanced biomaterials will enhance the effectiveness and biocompatibility of combinational cancer immunotherapy.

### 7.5 Combination of Sonodynamic Therapy and DC‐Mediated Immunotherapy

**Figure 18:**
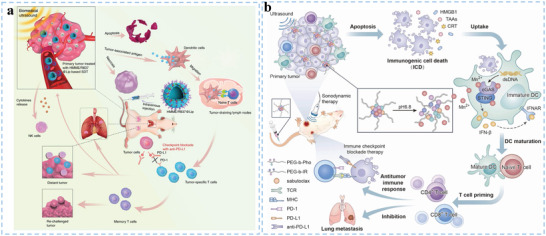
Biomaterials used for combined SDT‐immunotherapy. a) Schematic illustration of antitumor immune responses induced by combined noninvasive SDT with immune‐adjuvant‐contained nanosonosensitizers and ICBs for effective cancer immunotherapy. Reproduced with permission.^[^ ^244^ ^]^ Copyright 2019, Springer Nature. b) A schematic illustration shows that a phenolic nanoadjuvant (PIMS NPs) combines SDT with cGAS‐STING activation to promote significant maturation of DCs and enhance cancer immunotherapy. Reproduced with permission.^[^ ^245^ ^]^ Copyright 2022, Elsevier Ltd.

Currently, emerging sonodynamic therapy (SDT) has become a popular strategy due to its advantage of relatively deep tissue penetration depth.^[^ ^276^ ^]^ SDT modality for noninvasive cancer treatment has shown great potential, and can be utilized to activate sonosensitizers to generate ROS for inducing ICD under ultrasound, eliciting host antitumor immunological effects.^[^ ^277^ ^]^ Chen et al. developed a clinically approved ultrasound‐activated nanosonosensitizer (HMME/R837@Lip) for non‐invasive control of immunotherapy.^[^ ^244^ ^]^ The HMME/R837@Lip‐augmented SDT greatly activated DC maturation, arrested primary tumor progression, prevented tumor metastasis, and protected against tumor rechallenge (**Figure** **18**a). Tian et al. designed a phenolic nanoadjuvant (PEG‐IR‐Mn^2+^‐sabutoclax nanoparticles, PIMS NPs) for promoting antitumor immune responses, especially by facilitating the maturation of DCs.^[^ ^245^ ^]^ The PIMS NPs were designed to induce an SDT‐mediated ICD effect to kill tumors; therefore, dying tumor cells release DAMPs and antigens for the cross‐presentation of DCs. Mn^2+^ from PIMS NPs can activate the cGAS‐STING pathway of DCs to prime of cytotoxic T cells (Figure 18b). Thus, SDT is a non‐invasive and tumor‐directed cytotoxic therapeutic modality that has tremendous potential to induce DC maturation and antitumor immune responses.

## 8 Conclusion and Outlook

DC‐based immunotherapy has shown several exciting advantages, including specific CTL immunity, long‐term immunologic memory, and innate antigen presentation ability. In contrast to chimeric antigen receptor (CAR)‐T cells and artificial APCs, DCs can directly acquire specific antigen peptide information from TAAs via antigen presentation and processing.^[^ ^24^, ^27^ ^]^ Studies on DC‐based vaccines and preclinical strategies against cancer have been gradually translated into clinical practice.^[^ ^279^ ^]^ Recently, the increased understanding of immune regulation and the interplay between DCs and biomaterials has provided a scientifically solid foundation for the advancement of cancer immunotherapy. Advancements in interdisciplinary development have established that engineered biomaterials are feasible and safe tools for amplifying DC‐based tumor immunotherapy.^[^ ^280^ ^]^ Based on the desire to promote DC‐mediated immunotherapy, various biomaterials are continuously being studied and optimized to enhance DC function.^[^ ^281^ ^]^ Engineered biomaterials can not only be used as carriers for antigenic and immunostimulatory molecules but also suppress the negative effects of the TME.^[^ ^282^ ^]^ Therefore, it is important to design and optimize biomaterials to ensure perfect biocompatibility and long‐lasting immune‐stimulated efficacies for sustained DC activation. Different parameters of biomaterials, such as size, shape, targeting ligand, surface and mechanical properties, should be optimized to activate DCs and achieve effective co‐delivery of multiple antigenic and costimulatory components to further amplify DCs and relieve immune suppression.

It is worth noting that

(1) Further exploration of the contrastive efficiency and underlying mechanisms for biomaterials optimizing DC functions: As reported, many biomaterials can promote the immune cascade of DCs by delivering antigens and costimulators. In addition, some biomaterials themselves can be applied as adjuvants to promote DC maturation.^[^ ^283^ ^]^ The self‐adjuvanting properties of biomaterials for DC‐mediated antitumor therapy have unique advantages, such as simplification of DC vaccine composition, enhancement of the effects of other adjuvants, and improvement of the safety of DC vaccines. Both kinds of biomaterials can achieve certain activation effects; however, it is difficult to compare the DC activation efficiency of different biomaterials due to the lack of a unified control substance. In addition, the cellular signaling pathways of activated DCs involve multiple pathways, such as Toll‐like receptor, autophagy, JAK2‐STAT3, and cGAS‐STING.^[^ ^284^ ^]^ It has reported that Mn^2+^ can potentiate STING agonist activity and induce robust DC‐mediated antitumor T cell response with long‐term memory.^[^ ^285^ ^]^ However, current research tends to characterize the phenomena of DC activation and lacks exploration of the intrinsic biological mechanism. Therefore, the contrastive efficiency and underlying mechanisms of biomaterials need to be further studied.
(2) Challenges for in vivo delivery of biomaterials: Following administration of biomaterials into the body, they experience spontaneous opsonization and absorption of active biomolecules to form the so‐called “protein corona.”^[^ ^286^ ^]^ The biomaterials can be recognizable through corona‐cell communication and partly cleared by clearance pathways, such as the mononuclear phagocytic system.^[^ ^287^ ^]^ Engineering biomaterials with a specific “protein corona” can endow biomaterials with specific organotropic and cell‐specific targeting capability. In addition, biomaterials also interact with other barriers, such as endothelial walls constituting the blood vasculature, extracellular matrix, and cell membrane. Targeted delivery strategies for overcoming these barriers depend on the physical features of the biomaterials in terms of size, shape, surface charge, elasticity, *etc*. In addition, some novel‐designed nanosystems, which are characterized by a typical “core–shell” structure, can as programmed site‐specific delivery nanosystem to boost DC‐mediated anti‐tumor immunotherapy.^[^ ^288^ ^]^ These features also apparently affect the biodistribution and clearance of biomaterials. Thus, biomaterials need to optimize biodistribution in vivo, to ensure sufficient strength, speed, and duration of the DC immune response.
(3) Exploited emerging biomaterial forms: Novel formal designs of biomaterials need to be exploited, such as microneedles or 3D scaffolds. It is important for advancing the development of novel antigenic delivery systems for DCs. Some nanogels serve as multifunctional and constructed vectors formed by intramolecular cross‐linking to generate antigen delivery systems due to their satisfactory biocompatibility, bio‐responsiveness, high stability, and acceptable biocompatibility.^[^ ^289^ ^]^ Nanogels are 3D cross‐linked aqueous biomaterials that exhibit similar properties to natural tissues and can easily cope with shear forces and serum proteins in the bloodstream. Meanwhile, the large specific surface area can reduce or eliminate off‐target effects by adding stimuli‐responsive functional groups to promote targeting to specific DC subpopulations and conferring low immune‐related adverse events. Contributing to these biomaterials with novel forms, localized delivery of immune components to subcutaneous DCs can be sustained release. In addition, it has been reported the chirality of nanomaterials can influence their interaction with DCs and biological systems, further promoting the activation of NK cells and CTLs and their infiltration into tumor tissue.^[^ ^290^ ^]^ Thus, it is important to exploit emerging biomaterial forms for benefit functions of DCs.
(4) Challenges of the tumor‐suppressive immune microenvironment: Optimizing the efficiency of DC‐based immune therapy requires the activation of antitumor immune responses at multiple levels. Thus, novel biomaterials need to focus on collaborative functions that can be achieved by simultaneously activating DCs and reversing the immunosuppressive TME. In addition, tumors can be “hot” or “cold.”^[^ ^291^ ^]^ The immunological characteristics of “hot” and “cold” tumors are different and require different methods for targeting DC immunotherapy strategies. The application of biomaterials to achieve the conversion of “cold” tumors to “hot” tumors is a focus of much research. In addition, the TME significantly shapes the phenotype and function of DCs, which makes DCs dysfunctional and tolerogenic in antitumor response.^[^ ^278^ ^]^ Multi‐target biomaterials, which deliver signals able to enhance the function of multiple DC subsets may be needed to promote the efficacy and durability of the DC‐mediated immune response. Moreover, engineering multi‐target biomaterials with the ability to reshape immunosuppressive cells, including MDSCs, M2‐polarized TAMs, and Tregs, is promising for reversing the immunosuppressive TME to boost DC‐mediated immune responses.
(5) Future clinical translational perspective: With the progress of biomaterial science, more specific and effective regulation of DCs can be pursued. Although immunotherapy based on biomaterials has been deeply studied, only a very limited number of products have been applied from the laboratory to the clinic. Therefore, improving the clinical transformation potential of biomaterial‐based DC‐mediated immunotherapy is a major challenge. First, given the differences between human and preclinical models, the process of efficient antigen presentation by biomaterials still needs to be further improved to ensure sufficient activation of DCs both in vitro and in vivo, especially in large animal models. Moreover, several key prerequisites should also be considered before biomaterial‐based DC‐mediated immunotherapy can be widely commercialized, including high biosafety, scale‐up production, interbatch quality control, and long‐term stability. For biosafety reasons, one strategy is to use synthetic biomaterials with low toxicity. In addition, components from natural sources, such as cell membranes, extracellular vesicles, chitosan, or collagen, can be widely used to develop DC immunotherapy. However, compared with synthetic biomaterials, these natural‐derived biomaterials have difficulty in achieving large‐scale production, standard separation, and long‐term stability. Importantly, these biomaterials with moderate immune inducibility are more suitable for clinical transformation regardless of whether they are synthetic or natural. For manufacturing and quality control, simplified design is more likely to achieve scale‐up manufacturing with more controllable quality. Therefore, more efforts are needed to explore novel biomaterials with ideal function, controlled quality, and high clinical translational potential.

Biomaterials have exhibited great effects in DC‐based immunotherapy, including prophylactic, therapeutic, anti‐metastatic, and recurrence‐preventing effects. Studies on biomaterial‐enabled DC‐based vaccination are promising, as they aim to boost antigen‐specific antitumor immunity in patients in clinical treatment. In addition to the enhancement of potential therapeutic applications of DC immunity, recent advances in biomaterial‐based DC‐enhancing strategies have shown promise for the antitumor immune response. Explored emerging biomaterials, therefore, have facilitated the identification of crucial points that regulate DC activation. The application of functional biomaterials might also enable the future design of more effective therapeutics to regulate DCs and potentially solve the dilemma of tumor treatment. We hope that this review offers biomaterial engineers and immunologists insight into the immunological effects and mechanisms that build the foundation of a strong and durable DC immune response and bioengineering technologies that can be used to better control biomaterial performance.

## Conflict of Interest

The authors declare no conflict of interest.
